# Explainable machine learning framework with Granger-based temporal predictive validation for monitoring fiscal risk in national health insurance expenditure

**DOI:** 10.1371/journal.pone.0361904

**Published:** 2026-10-29

**Authors:** Gwangsu Lee

**Affiliations:** School of Computer Software, Daegu Catholic University, Gyeongsangbuk-do, South Korea; Guangdong Nephrotic Drug Engineering Technology Research Center, Institute of Consun Co. for Chinese Medicine in Kidney Diseases, CHINA

## Abstract

Rising health insurance expenditure (HIE) poses fiscal risks and challenges to the sustainability of public insurance systems. Despite growing interest in data-driven analysis, empirical evidence on the associations between macroeconomic, social, and financial conditions and national HIE remains limited, particularly across provider types. This study introduces an explainable machine-learning framework with temporal predictive validation to analyze and forecast HIE using 60 macro-financial and socioeconomic indicators. The framework is applied to three monthly expenditure series from Korea’s National Health Insurance system (2000–2023). Predictive performance and interpretability are assessed using two univariate autoregressive baselines, three regularized linear indicator-based benchmarks, and six nonlinear indicator-based ensemble models. LightGBM serves as the primary explanatory model, and SHapley Additive exPlanations (SHAP) quantifies indicator contributions and captures autoregressive persistence. Beyond associational insights, temporal predictive validation using lagged regression and Granger-type temporal predictive tests examines, within a prespecified exploratory lag window, whether SHAP-identified indicators show statistically supported exploratory lagged monitoring relevance. These results are interpreted as hypothesis-generating temporal monitoring evidence rather than as evidence of structural causal effects. Although persistence-based baselines achieved the strongest short-term forecasting accuracy, indicator-based models provided model-conditional explanatory information beyond autoregressive controls. The results indicate that shared macroeconomic pressures and provider-specific factors are associated with HIE predictions. The cost-of-living index is consistently associated with expenditure patterns across categories, whereas domestic demand and external conditions indicate provider-specific associations for medical institutions and pharmacies. Only a subset of predictors retained exploratory lagged monitoring support, underscoring the distinction between model-based predictive importance and hypothesis-generating monitoring signals. This integrated framework may inform transparent fiscal risk monitoring hypotheses and contextual review of public HIE.

## Introduction

Rapid advancements in artificial intelligence, big data, and intelligent information systems have accelerated the transition toward a data-driven society. Governments and industries increasingly employ large-scale statistical platforms—such as Korea’s IndexNuri system and the Bank of Korea’s Economic Statistics System—to support decision-making across economic, social, and financial domains [1,2]. Similarly, the Health Insurance Review and Assessment Service (HIRA) provides extensive health insurance expenditure (HIE) data generated from reimbursement reviews and service appropriateness assessments, offering substantial potential for risk-informed and finance-oriented analysis of public insurance expenditure systems [3]. In this study, public HIE refers to the total expenditure reimbursed by the Korean National Health Insurance system, as reported by HIRA.

Against this background, public HIE can be viewed not only as a healthcare outcome but also as a major component of public fiscal risk, warranting systematic analysis of its forecasting uncertainty and underlying risk indicators.

Although national statistical indicators have been widely applied to analyze and model macroeconomic conditions, business performance, industrial activity, and financial trends, prior studies have focused largely on business-oriented applications—including crude oil prices, stock indices, small and medium enterprise (SME) revenues, carbon emissions, and industry-specific sales [4–10]. These investigations commonly aim to (1) identify the most influential macroeconomic indicators for a target variable and (2) determine the optimal machine-learning (ML) algorithm based on dataset characteristics. However, despite their breadth, existing analyses rarely extend beyond business or industrial domains, leaving unresolved whether macroeconomic indicators have measurable predictive relevance in nonbusiness sectors such as healthcare, public health, and national social security systems.

In parallel, ML-based studies using health insurance data have made substantial progress in disease risk prediction, maternal and child health analysis, fraud detection, and insurance claims management [11–18]. However, these studies have focused predominantly on internal relationships within health insurance systems, such as predicting disease outcomes, determining insurance premiums, or detecting fraudulent behaviors. Importantly, existing research does not examine whether external macroeconomic environments are associated with national HIE predictions or whether these associations differ across healthcare provider types.

These limitations motivate the present study. National HIE accumulates nationwide through medical payments reimbursed by the National Health Insurance Service (NHIS) and HIRA, resulting in a large-scale system that may be sensitive to macroeconomic conditions. However, empirical evidence on the relationships between national statistical indicators and national HIE remains limited. Furthermore, no prior study has examined whether macro-financial predictive associations with national HIE differ between medical institutions and pharmacies, despite their distinct operational and functional roles in the public insurance system.

To address these limitations, this study investigates whether and how national statistical indicators—capturing major economic, social, and financial conditions—are associated with national HIE. Specifically, national HIE is disaggregated into medical institution and pharmacy components to examine whether expenditure-related monitoring indicators and predictive associations differ across provider types.

Accordingly, three research questions guide the analysis:

- Do macro-financial indicators provide predictive information for national HIE beyond intrinsic temporal persistence?
- Are these predictive associations heterogeneous across medical institution and pharmacy expenditures?
- Which SHapley Additive exPlanations (SHAP)-identified indicators show exploratory lagged monitoring support after Granger-type temporal screening and multiple-testing correction?

Consequently, this study aims to clarify the macro-financial sensitivity of national HIE and provide an interpretable framework for expenditure-risk monitoring rather than to outperform autoregressive benchmarks in short-term forecasting. Predictive performance is benchmarked using two univariate autoregressive baselines, three regularized linear indicator-based models [Ridge, least absolute shrinkage and selection operator (LASSO), and ElasticNet] serving as predictive benchmarks and model-dependence comparators, and six nonlinear ensemble models [eXtreme gradient boosting (XGBoost), light gradient boosting machine (LightGBM), categorical boosting (CatBoost), random forest (RF), adaptive boosting (AdaBoost), and gradient boosting] evaluated as nonlinear indicator-based candidates for explanatory analysis, all under time-ordered and rolling-origin evaluation. Indicator-based models are used to assess model-conditional explanatory value beyond expenditure persistence. The temporal validation step is then carried out to examine whether SHAP-identified indicators show statistically supported exploratory lagged monitoring relevance within the prespecified lag window.

This study has three contributions toward the literature on public insurance finance and risk analytics. First, it extends the application of macro-financial indicators beyond business contexts, providing evidence that national HIE is systematically associated with broader macroeconomic conditions after accounting for temporal persistence. Second, by distinguishing between medical institutions and pharmacies, it identifies provider-specific monitoring patterns in the predictive association between macroeconomic conditions and expenditure. Third, it proposes an explainable ML framework with temporal predictive validation that integrates SHAP-based interpretation with Granger-type temporal precedence testing, thereby distinguishing predictive importance from exploratory lagged monitoring support and supporting cautious policy-relevant interpretation in autoregressive expenditure environments.

## Related work

### Macroeconomic forecasting using statistical indicators

Macroeconomic indicators serve as fundamental inputs for forecasting various aspects of national and industrial activity. Prior studies commonly aim to identify (1) which indicators are most strongly associated with a target dependent variable and (2) which ML algorithm yields optimal predictive performance. Bharat et al. [7] analyzed crude oil prices using indicators such as the consumer price index, U.S. dollar, and gross domestic product and reported that the consumer price index and U.S. dollar were the most influential variables and that AutoRegressive Integrated Moving Average eXtended-based models were optimal predictors. Singh [8] applied decision trees, linear regression, support vector machine (SVM), AdaBoost, K-nearest neighbor (KNN), and other models to forecast the Nifty 50 index, identifying linear regression as the best-performing algorithm.

Other studies have applied ML techniques to domain-specific revenue prediction. Lee [4] analyzed 22 macroeconomic indicators related to inflation, employment, and interest rates to examine revenue changes among printing-related SMEs and reported that inflation-related indicators were most influential and that RF achieved the highest accuracy. Tan [9] investigated 44 macroeconomic indicators associated with carbon emissions, identifying coal consumption as the most important predictor and AdaBoost as the optimal algorithm. Additional studies have explored pharmacy sales [5], the National Association of Securities Dealers Automated Quotations index trends [10], and industry-specific corporate revenues [6], with consistent findings that (1) influential macroeconomic indicators differ by industry characteristics and (2) the optimal ML model depends on the data structure.

Across these works, a consistent methodological pattern emerges—macro-indicator selection, ML model comparison, and domain-specific identification of influential variables. However, existing studies remain largely confined to market- and industry-oriented sectors, with limited attention to public expenditure risk and insurance finance. Whether macroeconomic conditions are predictively associated with nonbusiness domains such as national insurance systems—particularly in terms of expenditure volatility and fiscal risk—remains insufficiently examined, representing a critical research gap addressed by the present study.

Table 1 summarizes ML studies that utilize macroeconomic indicators.

**Table 1:** ML studies utilizing macroeconomic indicators.

Study	Target Variable	Representative Indicators	Algorithms Compared	Best Algorithm
Bharat et al. [7]	Crude oil price	Consumer price index, U.S. dollar, gross domestic product	Vector autoregression, AutoRegressive Moving Average with Exogenous Inputs, linear regression, and AutoRegressive Integrated Moving Average	AutoRegressive Moving Average with Exogenous Inputs
Singh [8]	Nifty 50 index	Market & financial indicators	Decision trees, linear regression, SVM, AdaBoost, RF, and KNN	Linear Regression
Lee [4]	SME revenue	22 macroeconomic indicators	XGBoost, LightGBM, and RF	RF
Tan [9]	Carbon emissions	44 macroeconomic indicators	XGBoost, LightGBM, CatBoost, AdaBoost, RF	AdaBoost
Lee [5]	Pharmacy revenue	28 macroeconomic indicators	XGBoost, LightGBM, CatBoost, RF	RF
Zouaghia, et al. [10]	National Association of Securities Dealers Automated Quotations index	Financial market indicators	Gaussian naive Bayes, gradient boost, SVM, KNN, and RF	RF
Lee [6]	Industry revenues	58 national indicators	XGBoost, RF, CatBoost, AdaBoost, and gradient boosting	Gradient boosting

### Health insurance analytics using ML

ML applications in the health insurance sector have expanded rapidly, focusing primarily on prediction, classification, and risk assessment tasks. Joo et al. [11] used logistic regression, deep neural networks, RF, and LightGBM to predict cardiovascular disease risk in an Asian population and demonstrated that ML approaches outperform traditional clinical prediction models. Lee et al. [13] analyzed NHIS claims data to identify predictors of preterm birth and reported strong associations between maternal age, medication use, and birth outcomes. Additional works have predicted health insurance premiums [15], analyzed maternal heart disease and preterm birth [14], determined insurance claim eligibility [17], and developed fraud detection models using graph neural network, RF, XGBoost, and LightGBM [16,18]. Perinatal mortality has also been examined using ML algorithms [12].

Despite the growing body of literature on health insurance analytics and expenditure forecasting, prior studies have predominantly approached HIE from healthcare system–centric or utilization-based perspectives. Consequently, limited attention has been paid to HIE as a source of fiscal exposure and financial risk within public insurance systems. In particular, the conditional role of macro-financial and socioeconomic indicators in shaping expenditure volatility and risk dynamics remains underexplored from a financial management and risk-oriented perspective. To address this gap, this study reframes national HIE as a public financial risk management problem rather than a purely healthcare policy issue. By integrating macro-financial indicators into an explainable ML framework and explicitly controlling for expenditure persistence, the study provides new empirical evidence to support risk-informed fiscal monitoring and financial sustainability assessment in national insurance systems.

Table 2 summarizes ML studies utilizing health insurance data.

**Table 2:** ML studies utilizing health insurance data.

Study	Target Variable	Dataset Characteristics	Algorithms Compared	Best Algorithm
Joo et al. [11]	Cardiovascular disease risk	General health examination data (>510,000 individuals)	Logistic regression, RF, deep neural network, and LightGBM	Deep neural network and LightGBM
Lee et al. [13]	Preterm birth	NHIS claims data (405,586 mothers)	Logistic regression, RF, and artificial neural network	RF
Singh et al. [15]	Health insurance premiums	Health insurance market data	Linear regression, ensemble regression, and decision trees	Ensemble regression
Lee et al. [14]	Heart disease & preterm birth	NHIS claims data (174,926 mothers)	Logistic regression and RF	RF
Saraswat et al. [17]	Claim eligibility	Kaggle health insurance claims data	Logistic regression, naive Bayes, SVM, KNN, decision trees, and XGBoost	XGBoost
Yoo et al. [18]	Fraud detection	Kaggle insurance data	Graph neural network, logistic regression, RF, XGBoost, and LightGBM	Graph neural network
Nalluri et al. [16]	Fraud factors	U.S. Federal Health Insurance data	Decision trees, SVM, RF, and multi-layer perceptron	Multi-layer perceptron
Bogale et al. [12]	Perinatal mortality	Ethiopian Demographic Health Survey data	RF, CatBoost, and gradient boosting	Gradient boosting

### Methodological foundations: Trends in ML approaches

A synthesis of prior work across macroeconomic forecasting and health insurance analytics reveals several recurring methodological trends.

1. Comparative evaluation of multiple ML algorithms

Many studies compare ensemble-based models—such as XGBoost, LightGBM, CatBoost, RF, AdaBoost, and gradient boosting—to identify the optimal algorithm. Consistent findings show that algorithm performance depends heavily on data characteristics, including nonlinearity, feature heterogeneity, and temporal dependence.

2. Growing emphasis on interpretability and variable importance

Recent ML research increasingly incorporates interpretable artificial intelligence techniques—such as SHAP values and feature importance measures—to identify key predictors. This trend is particularly important in sectors where operational, regulatory, or policy decisions rely on empirical evidence. Both macroeconomic forecasting studies and health insurance analytics commonly seek to determine the most influential variables.

3. Recognition of temporal structures and lagged effects

Macroeconomic indicators and national HIE often exhibit temporal dependency, including seasonality, delayed effects, and structural shifts. Properly modeling these characteristics is essential for robust prediction and accurate identification of influential indicators.

4. Increasing use of temporal predictive validation and temporal robustness checks

While explainable artificial intelligence methods such as SHAP are widely used, recent studies emphasize validating whether identified predictors provide consistent lagged predictive information. Lag-based strategies, including lagged regression and Granger-type temporal predictive tests, are increasingly applied to distinguish predictive correlates from temporally supported monitoring indicators.

5. Implications for the present study

These methodological trends support the analytical design adopted in this study. First, because prior research has shown that no single ML algorithm consistently outperforms others across datasets, comparing multiple algorithms (XGBoost, LightGBM, CatBoost, RF, AdaBoost, and gradient boosting) is necessary to ensure model suitability.

Second, given the policy relevance of national HIE, identifying the national statistical indicators that are most strongly associated with national HIE predictions requires interpretable ML techniques such as SHAP.

Third, both macroeconomic indicators and national HIE follow monthly time-series patterns; thus, accounting for temporal dependence and potential lagged effects is essential for accurate modeling.

Fourth, temporal predictive validation is incorporated to test whether SHAP-identified indicators show statistically supported exploratory lagged monitoring relevance beyond autoregressive persistence.

Furthermore, unlike previous studies, the present work distinguishes between medical institutions and pharmacies—two provider types with distinct operational and structural characteristics. This design enables examination of whether macro-financial associations differ across provider types, addressing a previously unstudied dimension in the literature. Consequently, the analytical framework of this study is not only consistent with established methodological trends but also extends them to fill a significant research gap.

As shown in Tables 1 and 2, the optimal algorithm identified in prior studies varies substantially depending on dataset characteristics, including indicator composition and temporal structure. This variability suggests that model performance is determined less by the inherent superiority of a particular algorithm and more by the structural features of the data—such as nonlinearity, feature heterogeneity, and time dependence. In addition, although existing studies commonly identify key predictors and compare ML models, none have examined whether macroeconomic indicators are associated with national HIE predictions or whether these associations differ by provider type. These observations highlight the need for comprehensive algorithm comparison and variable importance analysis. Moreover, they underscore the contribution of the present study in bridging macroeconomic forecasting with HIE prediction.

## Research method

### Dataset

This study examines how national statistical indices representing economic, social, and financial conditions are associated with national HIE, a key component of the Korean health insurance system, within a system-level monitoring framework. From 746 national statistical indices, 72 indicators that provide monthly data were initially identified as candidates for analysis [1]. Among these, 12 indicators contained missing year–month values during the study period and were therefore excluded. The remaining 60 indicators with complete monthly observations from January 2000 to December 2023 were used as independent variables. These 60 indicators were categorized into the following groups: 9 employment-related, 10 global-related, 6 interest rate-related, 5 production-related, 7 raw material-related, 13 growth-related, 5 price-related, 3 housing-related, and 2 miscellaneous indices.

The final set of 60 indicators was determined a priori based on three criteria: monthly availability, complete coverage over the full study period, and conceptual relevance to macro-financial system monitoring. Rather than selecting indicators based on ex post predictive performance, the study retained a broad set of national indicators that could be plausibly associated with system-level public insurance expenditure monitoring, including labor-market conditions, external economic exposure, interest rates, production activity, commodity prices, growth dynamics, price levels, and housing conditions. This design was intended to preserve comprehensive macro-financial coverage while avoiding outcome-driven indicator selection or imputation-based bias.

The detailed descriptive statistics for these 60 national statistical indicators have been moved to the appendix to maintain clarity and conciseness in the main text. Readers may refer to S1 Appendix A (Table A1) for the complete statistics, including the minimum, maximum, mean, and standard deviation values for each indicator.

The dependent variable in this study is national HIE. National HIE data were obtained from HIRA, which publishes monthly HIE statistics for 101,762 medical care institutions nationwide [19]. Among these institutions, 77,055 were medical institutions and 24,707 were pharmacies. The expenses were compiled from claims submitted by medical institutions and pharmacies nationwide in accordance with the National Health Insurance Act.

Table 3 presents the number of medical care institutions and descriptive statistics for national HIE categorized into medical institutions and pharmacies.

**Table 3:** Descriptive statistics by medical care institution.

Medical Care Institution	Number of Medical Care Institutions	Min	Max	Mean	Std. Dev.
Medical Institution	77,055	806,436,930	9,386,445,608	3,347,702,502.51	1,948,891,375.46
Pharmacy	24,707	20,063,839	2,014,689,630	986,655,829.00	457,634,127.94
Total	101,762	826,500,769	11,324,317,237	4,334,358,331.51	2,393,530,237.40

Using these institution-level monthly statistics, this study constructed national-level monthly expenditure series for each provider group. Specifically, for every month in the study period, national HIE was aggregated to obtain (1) the total national HIE for all institutions, (2) the national total for medical institutions only, and (3) the national total for pharmacies only. Accordingly, each observation in the modeling dataset represents one month of nationwide total expenditure for a given provider group, matched to the corresponding monthly values of the 60 national statistical indicators via the common year–month identifier.

The modeling dataset is constructed at the national monthly aggregate level; hence, the unit of analysis is the month-level national expenditure series rather than individual patients, individual medical care institutions, regions, or claims-level observations. Therefore, the empirical design should be interpreted as an ecological, system-level monitoring framework. The results do not support inference about individual-level behavior, institution-level causal mechanisms, regional heterogeneity, or counterfactual policy effects. Therefore, potential aggregation bias and omitted system-level confounding are considered in the interpretation of the findings.

The decision to use monthly data is consistent with both the structure of the source data and the objectives of this study. First, the HIRA statistics are compiled and disclosed monthly, reflecting the natural billing and reimbursement cycle of the national health insurance system. Second, the national statistical indicators used as independent variables are also provided at a monthly frequency, enabling precise time alignment between macroeconomic conditions and national HIE without aggregation-induced information loss. Third, monthly data enable the models to capture short-term fluctuations, seasonality, and potential lagged effects of macroeconomic conditions on national HIE, which would be difficult to identify using only quarterly or annual aggregates.

With respect to feature selection, the 12 indicators with missing values were removed for data-quality reasons, and no additional filter-, wrapper-, or embedded feature-selection methods were applied before model training. The remaining 60 indicators were retained to preserve broad macro-financial coverage and avoid prematurely excluding potentially relevant monitoring information. Indicator importance was evaluated post hoc using SHAP, followed by selective temporal predictive validation. This design should be interpreted as post-hoc importance screening rather than premodel variable elimination or formal dimensionality reduction. As the indicators are correlated, SHAP rankings may reflect attribution dispersion across related predictor clusters; therefore, individual rankings are interpreted as model-conditional screening signals rather than definitive variable-selection results.

### ML analysis

This study employs a comparative modeling framework comprising two univariate autoregressive baseline models, three regularized linear indicator-based benchmark models, and six nonlinear indicator-based ensemble ML models to analyze national HIE. The proposed design explicitly distinguishes between forecasting accuracy and policy-relevant explanations, which serve different analytical objectives in highly autoregressive time-series settings.

#### Univariate autoregressive baseline models.

The two univariate autoregressive baseline models considered were the persistence (t − 1) and same-month-last-year (t − 12) models. These models capture short-term and seasonal autoregressive structures, respectively. However, they do not incorporate national statistical indicators; therefore, they serve as purely time-dependent forecasting benchmarks.

#### Regularized linear indicator-based benchmark models.

Ridge regression, LASSO regression, and ElasticNet regression were included as regularized linear indicator-based benchmark models. These models use the same explanatory-variable structure as the indicator-based ML models and provide coefficient-based linear comparators for evaluating predictive performance, shrinkage behavior, and model dependence under correlated macro-financial predictors.

#### Nonlinear indicator-based ensemble ML models.

To enable policy-relevant explanations of national HIE, this study analyzes six nonlinear indicator-based ensemble ML models: XGBoost, LightGBM, AdaBoost, gradient boosting, CatBoost, and RF. These models incorporate national statistical indicators and are used to model nonlinear explanatory patterns beyond purely autoregressive baselines.

The six nonlinear models were included as representative tree-based ensemble comparators. RF represents a bagging-based approach, whereas AdaBoost, gradient boosting, XGBoost, LightGBM, and CatBoost represent boosting-based approaches [20]. These models were compared under a unified time-series validation framework to evaluate nonlinear indicator-based specifications. As the national statistical indicators used in this study are predominantly numerical, CatBoost was retained as a robustness benchmark rather than as a categorical-specialized model. The study focuses on comparative forecasting performance and SHAP-based interpretation rather than algorithm development.

#### Role differentiation among model groups.

Univariate autoregressive baseline models were used as purely time-dependent forecasting benchmarks, whereas indicator-based models were used to evaluate whether national statistical indicators added model-conditional explanatory value beyond expenditure persistence. Among the indicator-based models, Ridge, LASSO, and ElasticNet were retained as regularized linear predictive benchmarks and model-dependence comparators, whereas LightGBM was used as the primary nonlinear explanatory model for SHAP-based interpretation. SHAP-selected indicators were subsequently evaluated using Granger-type temporal predictive validation to assess exploratory lagged monitoring relevance after stationarity transformation and multiple-testing correction.

The nonlinear ensemble models require distinct algorithm-specific implementations. Table 4 presents the software packages used to implement the ensemble ML algorithms used in the study.

**Table 4:** Software packages used by ensemble ML algorithms.

Algorithm	Package
XGBoost	XGBRegressor
LightGBM	LGBMRegressor
AdaBoost	AdaBoostRegressor
Gradient boost	GradientBoostingRegressor
CatBoost	CatBoostRegressor
RF	RandomForestRegressor

#### Research analysis procedure.

Building on the conceptual distinction between baseline and indicator-based models, this subsection describes the end-to-end analytical procedure employed in this study, encompassing data preprocessing, model training, validation, and evaluation (Fig 1).

**Fig 1:**
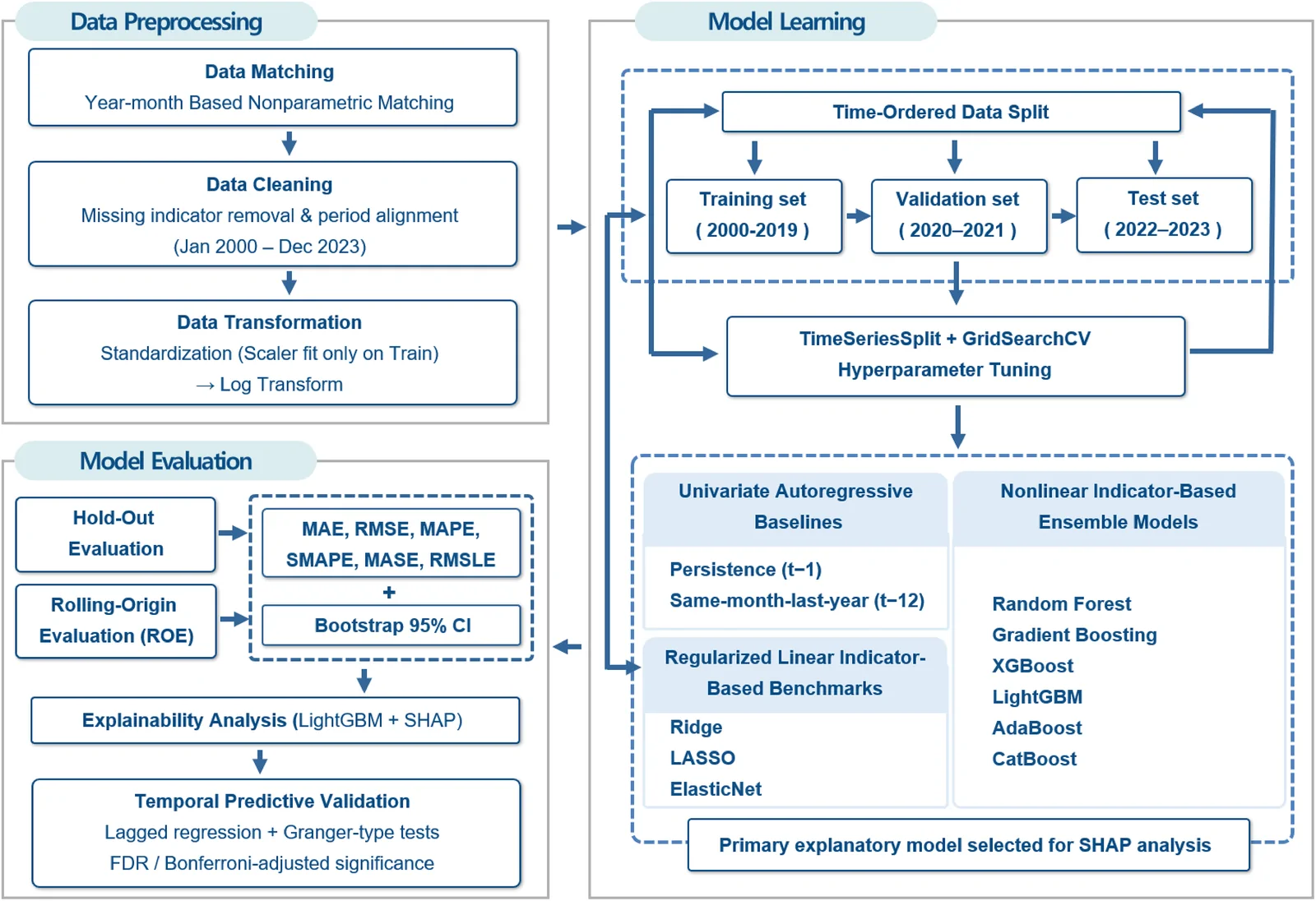
Research analysis procedure.

The analysis procedure comprised three main steps: data preprocessing, model learning, and model evaluation (Fig 1). Each step is performed sequentially, with the initial phase, data preprocessing, preparing the collected datasets for analysis through data matching, purification, and transformation.

The first step, data matching, involves integrating different datasets using common identifiers. Data matching can be classified as either exact or statistical matching, depending on whether unique identifiers are available in all datasets. Exact matching directly links records with identical unique identifiers, whereas statistical matching integrates datasets using shared variables when such identifiers are unavailable [21,22]. Statistical matching can be further classified into parametric and nonparametric approaches. In this study, nonparametric matching was appropriate because the HIE data and the national statistical indicators shared only one common variable: the year–month identifier.

Therefore, the 60 national statistical indicators were integrated as independent variables with the national HIE series aggregated from 101,762 medical care institutions as dependent variables using year–month-based nonparametric matching (Fig 2). The independent-variable dataset comprised year–month values and corresponding national statistical indicator values, whereas the dependent-variable dataset comprised year–month values and corresponding national HIE outcomes. As the datasets were linked only through the common year–month identifier, without individual-, institution-, regional-, or claims-level identifiers, the resulting dataset comprised national monthly aggregate observations matched across expenditure outcomes and macro-financial indicators.

**Fig 2:**
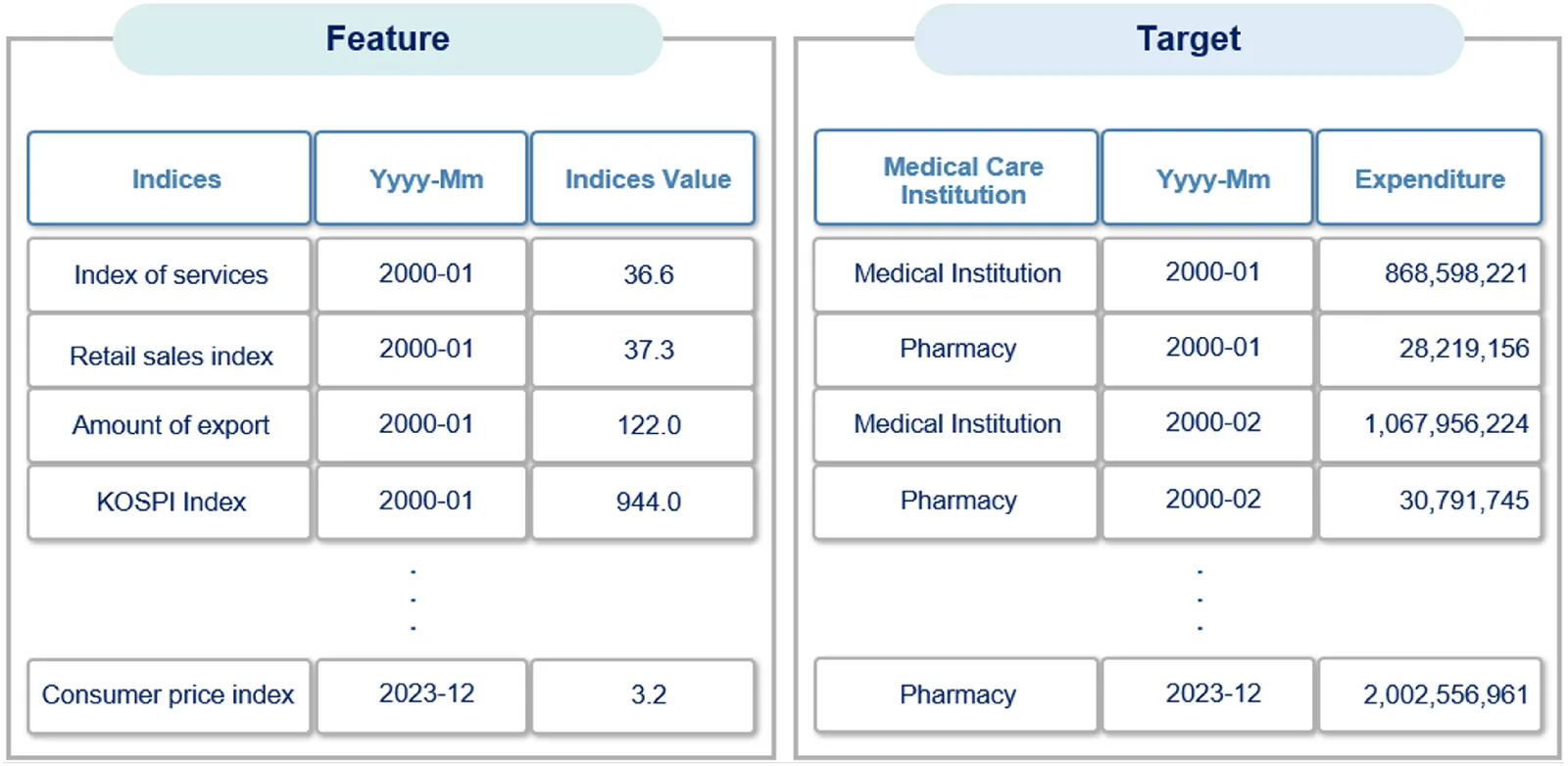
Feature data and target data layout.

Second, data cleansing involves extracting and integrating only the necessary data from the collected dataset to prepare the information for analysis. This process includes the removal of duplicate records, the filtering of outliers, and the treatment of missing values. An examination of the matched dataset revealed that the independent variables had different periods of data availability because of the differing characteristics of each index. Additionally, certain indices, particularly those recorded manually before the launch of the IndexNuri and economic statistics systems, lacked corresponding year‒month indicator values. Similarly, the dependent variable—national HIE—was recorded starting from the implementation of the National Health Insurance Act in 2000.

To ensure data quality and consistency, entries with missing year‒month values for either the independent or dependent variables were removed. Specifically, 12 out of 72 indicators containing missing values were excluded; the remaining 60 indicators were used as independent variables. Furthermore, the analysis period was limited to the common timeframe shared by both datasets, spanning from January 2000 to December 2023.

Third, data transformation involves modifying the format or structure of the data to increase their suitability for analysis. Techniques such as normalization and log scaling were applied based on the distribution characteristics of the data.

An analysis of the matched dataset revealed discrepancies in the units and ranges of both the independent and dependent variables, depending on the characteristics of the statistical indicators. In particular, standardizing the units and ranges of the independent indicators was essential for ensuring comparable model inputs and for evaluating variable importance and model performance. Feature scaling was applied to address these issues and align the variables to a consistent range; logarithmic transformation was applied not uniformly to all variables but where appropriate to reduce scale imbalance and skewness [6].

Prior to time-series modeling and temporal predictive validation, the stationarity properties of the HIE series and national statistical indicators were assessed using both the augmented Dickey–Fuller (ADF) test and the Kwiatkowski–Phillips–Schmidt–Shin (KPSS) test. The ADF test evaluates the null hypothesis of a unit root, whereas the KPSS test evaluates the null hypothesis of stationarity. The three HIE series were tested in levels and after first-order logarithmic differencing. Variables that remained nonstationary in levels were transformed before Granger-type temporal predictive validation. Standardization was used only for model training and was not considered a stationarity-inducing transformation. The main stationarity results for the three HIE series are reported in Table 5; the full results for the national statistical indicators are reported in S4 Appendix D.

**Table 5:** Stationarity diagnostics for the three HIE series based on ADF and KPSS tests.

Category	ADF		KPSS		Stationarity	Treatment
	Statistic	p-value	Statistic	p-value		
Total HIE (level)	3.09501	1.00000	2.61645	0.01000	Nonstationary	First-order logarithmic differencing
Total HIE (log-diff)	−5.23370	7.50E-06	0.40803	0.07369	Stationary	Used for Granger-type temporal predictive validation
Medical institution HIE (level)	3.74717	1.00000	2.59739	0.01000	Nonstationary	First-order logarithmic differencing
Medical institution HIE (log-diff)	−5.26574	6.44E-06	0.34797	0.09958	Stationary	Used for Granger-type temporal predictive validation
Pharmacy HIE (level)	1.58048	0.99780	2.62375	0.01000	Nonstationary	First-order logarithmic differencing
Pharmacy HIE (log-diff)	−8.15730	9.34E-13	0.34238	0.10000	Stationary	Used for Granger-type temporal predictive validation

Note: ADF = augmented Dickey–Fuller test; KPSS = Kwiatkowski–Phillips–Schmidt–Shin test. ADF null hypothesis: unit root (nonstationary). KPSS null hypothesis: stationarity. Log-diff = first-order logarithmic differencing. Significance level: α = 0.05.

The model learning step was designed to ensure temporal validity, prevent information leakage, and establish a rigorous and reproducible forecasting framework. To preserve the chronological structure inherent in the monthly time-series data, the full dataset was divided into three nonoverlapping temporal segments: a training period (January 2000–December 2019), a validation period (January 2020–December 2021), and a test period (January 2022–December 2023). This time-ordered split strategy prevents future information from influencing model training and more accurately reflects real-world forecasting conditions.

All feature scaling operations were performed using parameters estimated exclusively from the training period. Specifically, StandardScaler was fitted on the training set and subsequently applied to the validation and test sets, thereby avoiding leakage arising from normalization across temporal boundaries.

Hyperparameter optimization was conducted within the training period using TimeSeriesSplit with five folds. In contrast to conventional k-fold cross-validation, TimeSeriesSplit expands the training window sequentially while maintaining temporal order, enabling model selection without temporal information leakage. The hyperparameter search space for each algorithm (summarized in Table 6) was systematically explored using GridSearchCV. After the optimal hyperparameter combination was identified, the model was refitted during the full training period before out-of-sample evaluation. The final optimized hyperparameters for the selected LightGBM explanatory models are reported in S2 Appendix B, Table B7, to improve reproducibility.

**Table 6:** Hyperparameter search spaces used for model tuning.

Algorithm	Hyperparameter Values
XGBoost	n_estimators: [100, 200, 300, 400, 500, 1000] learning_rate: [0.01, 0.05, 0.1, 0.2, 0.3, 0.4, 0.5] max_depth: [2–8] gamma: [0, 0.1, 0.2, 0.3, 0.4, 0.5, 1] min_child_weight: [1–5] subsample: [0.5, 0.7, 0.8, 0.9, 1] colsample_bytree: [0.5, 0.6, 0.7, 0.8, 0.9, 1] reg_lambda: [1,3,5,7] random_state: [19]
LightGBM	n_estimators: [100, 200, 300, 400, 500, 1000] learning_rate: [0.01, 0.05, 0.1, 0.2, 0.3, 0.4, 0.5] num_leaves: [20,31,50,100,150] max_depth: [−1, 3, 4, 5, 6, 7] min_child_samples: [5,10,20,50,100] subsample: [0.5, 0.6, 0.7, 0.8, 0.9, 1] colsample_bytree: [0.5, 0.6, 0.7, 0.8, 0.9, 1] seed: [19]
AdaBoost	n_estimators: [100, 200, 300, 400, 500, 1000] learning_rate: [0.01, 0.05, 0.1, 0.2, 0.3, 0.4, 0.5] loss: [“linear,” “square,” “exponential”] max_depth of base_estimator(DecisionTreeRegressor): [1–4] random_state: [19]
Gradient boost	n_estimators: [100, 200, 300, 400, 500, 1000] learning_rate: [0.01, 0.05, 0.1, 0.2, 0.3, 0.4, 0.5] max_depth: [2–8] min_samples_leaf: [1,2,4,8,12,18] min_samples_split: [2,4,8,16,20] subsample: [0.5, 0.7, 0.8, 0.9, 1] random_state: [19]
CatBoost	iterations: [100, 200, 300, 400, 500, 1000] learning_rate: [0.01, 0.05, 0.1, 0.2, 0.3, 0.5, 0.7, 1.0] depth: [2,4,6,8,10,12,14,16] l2_leaf_reg: [1,3,5,7] subsample: [0.5, 0.7, 0.8, 0.9, 1] rsm: [0.5, 0.7, 0.8, 0.9, 1.0] random_seed: [19]
RF	n_estimators: [100, 200, 300, 400, 500, 1000] max_depth: [6,8,10,12] min_samples_leaf: [1,2,4,8,12,18] min_samples_split: [2,4,8,16,20] random_state: [19]

Model performance was evaluated on both the validation and test periods using a comprehensive set of error metrics, including the MAE, RMSE, MAPE, SMAPE, MASE, and RMSLE. The statistical uncertainty of each metric was quantified by computing 1,000-iteration bootstrap confidence intervals (95% CI) based on resampled residuals.

In addition to the hold-out validation and test assessments, the robustness of the forecasting procedure was further examined through a rolling-origin evaluation (ROE) with an expanding-window design. For each time step from 2020 to 2023, the model was retrained on all available historical observations up to that point and used to generate a one-step-ahead forecast. The performance statistics derived from the ROE were also accompanied by bootstrap-based 95% confidence intervals, providing an additional layer of reliability assessment.

The predictive performance of each ML algorithm was evaluated by calculating regression performance metrics, including MAE, RMSE, MAPE, SMAPE, MASE, and RMSLE, for both the validation and test periods. The following equations define these metrics in terms of the observed and predicted values at time *i*, denoted by ${\text{y}}_{\text{i}}$ and $\hat{y_{\text{i}}}$, respectively; the total number of observations, *n*; and the naive scaling lag parameter, *m*. For MASE, *m* was set to 1 to use a one-step nonseasonal naive scaling benchmark.

The MAE is calculated by converting the differences between the predicted value $\hat{y_{\text{i}}}$ and the actual value ${\text{y}}_{\text{i}}$ into absolute values and then taking the average, as follows:


$$\begin{array}{c} \text{MAE}\;=\;\frac{\text{1}}{\text{n}}\;\sum_{\text{i}=\text{1}}^{\text{n}}\;|{\text{y}}_{\text{i}}-\hat{y_{\text{i}}}| \end{array}$$ (1)

The RMSE is calculated by taking the square root of the average of the squared differences between the predicted value $\hat{y_{\text{i}}}$ and the actual value ${\text{y}}_{\text{i}}$ as follows:


$$\begin{array}{c} \text{RMSE}=\sqrt{\frac{\text{1}}{\text{n}}\sum_{\text{i}=\text{1}}^{\text{n}}({\text{y}}_{\text{i}}-\hat{y_{\text{i}}}{)}^{\text{2}}} \end{array}$$ (2)

The MAPE is calculated by expressing the prediction error as a percentage of the actual value and then taking the average, as follows:


$$\begin{array}{c} \text{MAPE}\;=\;\frac{\text{100}}{\text{n}}\;\sum_{\text{i}=\text{1}}^{\text{n}}\;|\frac{{\text{y}}_{\text{i}}-\hat{y_{\text{i}}}}{{\text{y}}_{\text{i}}}| \end{array}$$ (3)

The SMAPE addresses the asymmetry of the MAPE by normalizing the absolute error using the average magnitude of the actual and predicted values. It is calculated as follows:


$$\begin{array}{c} \text{SMAPE}\;=\;\frac{\text{100}}{\text{n}}\;\sum_{\text{i}=\text{1}}^{\text{n}}\;\frac{\left|{\text{y}}_{\text{i}}-\hat{y_{\text{i}}}\right|}{(\left|{\text{y}}_{\text{i}}\right|+\left|\hat{y_{\text{i}}}\right|)/\text{2}} \end{array}$$ (4)

The MASE is a scale-independent metric that compares the forecasting error of the model to that of a naive benchmark and is calculated as follows:


$$\begin{array}{c} \text{MASE}\;=\frac{\;\frac{\text{1}}{\text{n}}\;\sum_{\text{i}=\text{1}}^{\text{n}}\;|{\text{y}}_{\text{i}}-\hat{y_{\text{i}}}|}{\;\frac{\text{1}}{\text{n}-\text{m}}\;\sum_{\text{i}=\text{m}+\text{1}}^{\text{n}}\;|{\text{y}}_{\text{i}}-{\text{y}}_{\text{i}-\text{m}}|} \end{array}$$ (5)

The RMSLE applies a logarithmic transformation to reduce the influence of large values and emphasizes proportional rather than absolute differences. It is calculated as follows:


$$\begin{array}{c} \text{RMSLE}=\sqrt{\frac{\text{1}}{\text{n}}\sum_{\text{i}=\text{1}}^{\text{n}}(\text{ln}(\text{1}+\hat{y_{\text{i}}}{)-\text{ln}(\text{1}+{\text{y}}_{\text{i}}))}^{\text{2}}} \end{array}$$ (6)

The closer the values of the MAE, RMSE, MAPE, SMAPE, MASE, and RMSLE are to zero, the higher is the predictive accuracy. Accordingly, the ML algorithm with the smallest error values across these evaluation metrics is considered more effective for forecasting national HIE [6,8].

The model evaluation and interpretation workflow comprised four sequential components: forecasting-performance comparison, explanatory-model interpretation, provider-type comparative assessment, and temporal predictive validation.

First, forecasting performance was compared across baseline, regularized linear, and nonlinear indicator-based models using MAE, RMSE, MAPE, SMAPE, MASE, and RMSLE during the validation and test periods. The same metrics were also evaluated using ROE with an expanding-window design.

Second, for model interpretation, LightGBM was selected separately as the primary nonlinear explanatory model for SHAP-based analysis, rather than as the best forecasting model. Using this LightGBM model, SHAP values were subsequently used to quantify the model-conditional contribution of each national statistical indicator to HIE predictions. SHAP provides a principled attribution of feature contributions within the fitted model, enabling identification of indicators with high model-conditional predictive importance while supporting monitoring-oriented interpretation. Notably, under conditions of multicollinearity among the 60 national statistical indicators, SHAP values may distribute predictive importance across correlated predictors, reflecting the joint contribution of a predictor cluster rather than the isolated effect of any single variable. To address this, SHAP-based rankings were used as screening signals for subsequent temporal predictive validation rather than as definitive variable-selection criteria. Their model dependence was also examined through a sensitivity analysis comparing LightGBM-SHAP rankings with ElasticNet-based standardized coefficient rankings, as reported in S3 Appendix C.

To quantitatively assess predictor multicollinearity among the national statistical indicators, we additionally computed pairwise Pearson correlation coefficients and variance inflation factors (VIFs) using the training-period predictor matrix after log1p preprocessing. Autoregressive lag features were excluded so that the diagnostics would only reflect multicollinearity among the 60 national statistical indicators. The pairwise correlation analysis comprised 1,770 indicator pairs from 228 training-period observations and was used to identify highly correlated indicator pairs and broader correlation patterns among the predictors. VIF diagnostics were calculated for all 60 indicators to quantify the extent to which each indicator could be linearly explained by the remaining indicators. These diagnostics were not used for feature elimination, because the study aimed to preserve broad macro-financial coverage; instead, they were used to support the interpretation of SHAP rankings as model-conditional and potentially cluster-level monitoring signals. The multicollinearity diagnostics are reported in S3 Appendix C, Tables C6 and C7.

Third, to examine whether HIE-associated monitoring indicators differ between medical institutions and pharmacies, performance metrics and SHAP-based variable contributions were analyzed separately for each provider type. This comparative evaluation revealed provider-specific differences in the macroeconomic indicators associated with expenditure predictions.

Finally, temporal predictive validation was conducted to assess whether SHAP-identified indicators showed statistically supported exploratory lagged monitoring relevance for expenditure dynamics. Lagged regression and Granger-type temporal predictive tests were used on key indicators to distinguish model-based predictive correlations from statistically supported exploratory lagged monitoring relevance.

### Temporal predictive validation framework

Although SHAP-based feature attribution identifies indicators that are conditionally associated with model predictions, it does not establish whether these variables provide additional lagged predictive information beyond expenditure persistence. To complement associational interpretation, a temporal predictive validation framework is introduced to assess whether SHAP-identified indicators show exploratory lagged monitoring support in a time-series context.

For each expenditure series (total, medical institution, and pharmacy), the top-ranked indicators derived from SHAP analysis were subjected to Granger-type temporal predictive testing. To limit the multiple-testing burden, Granger-type temporal validation was conducted exclusively on SHAP-selected indicators rather than on all 60 candidate variables. This two-stage procedure—SHAP screening followed by temporal validation—reduces the number of simultaneous hypothesis tests from 60 to the top-ranked subset per expenditure category, thereby reducing the multiple-testing burden before applying false discovery rate (FDR) and Bonferroni adjustments. The analysis explicitly controlled for autoregressive persistence by including lagged dependent variables, thereby isolating the incremental predictive contribution of each macro-financial indicator.

Formally, for each indicator ${\text{X}}_{\text{t}}$ and expenditure series ${\text{Y}}_{\text{t}}$, the following lag-augmented regression model was estimated [23]:


$$\begin{array}{c} {\text{Y}}_{\text{t}}=\alpha +\sum_{\text{i}=\text{1}}^{\text{p}}{\beta_{\text{i}}\text{Y}}_{\text{t}-\text{i}}\;+\sum_{\text{j}=\text{1}}^{\text{q}}{\gamma_{\text{j}}\text{X}}_{\text{t}-\text{j}}\;+\epsilon_{\text{t}} \end{array}$$ (7)

where p denotes the number of autoregressive lags of the dependent variable and q denotes the number of lags of the candidate indicator. The joint significance of the coefficients $\gamma_{\text{j}}$ was evaluated using an F-test within a Granger-type temporal predictive testing framework [23,24].

Temporal predictive validation was conducted using a combination of lagged regression models and Granger-type temporal predictive tests. The null hypothesis states that lagged values of an indicator do not improve the prediction of expenditure beyond its own historical values:


$$\begin{array}{c} {\text{H}}_{\text{o}}:\gamma_{\text{1}}=\gamma_{\text{2}}=\ldots =\gamma_{\text{q}}=\text{0} \end{array}$$ (8)

To reduce the risk of spurious temporal associations, Granger-type temporal predictive validation was conducted using stationarity-transformed series. Based on the stationarity diagnostics, nonnegative HIE and indicator series were transformed using first-order log1p differences, whereas variables unsuitable for logarithmic differencing were transformed using first differences. When transformed series remained mixed or inconclusive according to the combined ADF and KPSS diagnostics, the corresponding Granger-type results were retained for supplementary screening but interpreted cautiously as stationarity-sensitive temporal monitoring evidence rather than as fully robust temporal predictive evidence. The maximum lag window was prespecified as 12 months, reflecting the annual seasonality of monthly HIE data. For each SHAP-selected indicator, Granger-type tests were estimated for lags 1–12, and the lag with the smallest SSR_F-test p-value was reported as the exploratory selected lag. This approach was used to identify the strongest lagged monitoring signal within the prespecified annual window rather than to perform confirmatory lag-order selection. Lag selection based on the Akaike information criterion or the Bayesian information criterion was not used as the primary procedure. The objective of the temporal validation step was to screen whether SHAP-identified indicators provided additional lagged monitoring information beyond autoregressive expenditure persistence, rather than to identify the best-fitting time-series model for each indicator. Applying information-criterion-based lag selection separately to each indicator and expenditure category could also produce heterogeneous lag structures that are less comparable across provider types. Similarly, a single prespecified common lag was not used as the primary specification because macro-financial indicators may operate over different short- and medium-term horizons. Instead, the 1–12-month window was retained as an exploratory annual screening range, and a lag-search-adjusted sensitivity analysis was added to evaluate the robustness of the reported minimum-p-value results. Specifically, for each SHAP-selected indicator, the minimum p-value selected across lags 1–12 was additionally adjusted using a conservative Bonferroni correction across the 12 candidate lags. These lag-search-adjusted p-values were then used for supplementary FDR and Bonferroni correction within each expenditure category-specific SHAP-selected test family. The results are reported in S2 Appendix B, Table B6, and are interpreted as sensitivity evidence rather than as confirmatory evidence of robust lagged predictive relationships or causal effects. Residual autocorrelation diagnostics were subsequently conducted using the Ljung–Box test for the lagged regression models underlying the Granger-type temporal predictive validation, as reported in S2 Appendix B, Table B5. Residual autocorrelation diagnostics for the selected LightGBM explanatory models are also reported in S2 Appendix B, Table B4.

Notably, Granger-type testing establishes predictive temporal precedence, rather than structural causation. Because the original predictor set contains 60 correlated macroeconomic indicators with strong autocorrelation, two limitations require explicit consideration: collinearity may inflate or deflate individual F-statistics, and multiple testing across SHAP-selected indicators may increase the risk of false positives. In the main temporal predictive validation table, all reported raw p-values were accompanied by Benjamini–Hochberg FDR-adjusted p-values and Bonferroni-adjusted p-values calculated within each expenditure category-specific SHAP-selected test family. Because these main-table adjustments do not, by themselves, correct for the exploratory lag search across candidate lag specifications, the lag-search-adjusted sensitivity analysis reported in S2 Appendix B, Table B6 was added as a supplementary robustness check. Accordingly, the Granger-type results should be interpreted as exploratory temporal monitoring evidence rather than confirmatory evidence of robust lagged predictive relationships or causal effects. Only indicators with FDR-adjusted p-values below α = 0.05 were classified as indicators with exploratory lagged monitoring support, whereas indicators also surviving Bonferroni correction were interpreted as having stronger statistical support within the SHAP-selected temporal screening family. In addition, the inclusion of lagged dependent variables as autoregressive controls, although necessary to estimate the incremental predictive contribution of macro-financial indicators, may reduce the statistical power of Granger-type tests when autoregressive persistence dominates.

Therefore, these results should be interpreted as statistically supported exploratory temporal monitoring evidence rather than as evidence of structural causal mechanisms. By integrating SHAP-based attribution with Granger-type temporal predictive validation, the framework enables a more cautious interpretation of model outputs by distinguishing model-based predictive importance from statistically supported exploratory lagged monitoring relevance, while enhancing methodological transparency and statistical rigor.

### Software environment

All analyses were conducted in Python (version 3.11.5) in a Windows 11 environment via the Anaconda distribution. ML models were implemented with the following libraries: scikit-learn (version 1.3.0), XGBoost (version 1.7.3), LightGBM (version 4.3.0), and CatBoost (version 1.2.5). RF, gradient boosting, and AdaBoost models were implemented via the scikit-learn ensemble module.

To ensure full reproducibility, all algorithms were executed with a fixed random seed (19), including scikit-learn’s random_state, XGBoost’s random_state, LightGBM’s seed, and CatBoost’s random_seed.

Hyperparameter optimization was performed using the GridSearchCV function in scikit-learn with a time-series-aware cross-validation strategy (TimeSeriesSplit). All computations were executed on a personal computer equipped with an Intel Ultra 7 CPU and 32 GB of RAM.

## Results

### Overall forecasting performance

This section presents the out-of-sample forecasting performance of the baseline and ML models for the three HIE series during the test period (2022–2023). The model performance was assessed using RMSE and MAE as the primary metrics, following the best practices for time-series evaluation. Additional accuracy measures, 95% bootstrap confidence intervals, and residual autocorrelation diagnostics are reported in S2 Appendix B (Tables B1–B4). The Ljung–Box test showed no significant residual autocorrelation for either the total or medical institution HIE. For pharmacy HIE, residual autocorrelation was detected in the 2022–2023 holdout test residuals, but not in the ROE residuals, which suggests that the pharmacy test-period results should be cautiously interpreted.

Table 7 compares the best-performing univariate autoregressive baseline model with the best-performing nonlinear indicator-based ensemble model for each expenditure component. Across all three series, the persistence model (t-1) achieved the lowest RMSE, confirming the strong temporal dependence of the monthly national HIE.

**Table 7:** Summary of the out-of-sample forecasting performance for the test period (2022–2023).

Category	Best univariate autoregressive baseline	RMSE (Test mean)	MAE (Test mean)	Best nonlinear indicator-based ensemble model	RMSE (Test mean)	MAE (Test mean)
Total HIE	Persistence (t-1)	**0.09297**	0.06304	LightGBM	0.24231	0.22665
Medical institution HIE	Persistence (t-1)	**0.11088**	0.07711	Gradient boosting	0.19807	0.17677
Pharmacy HIE	Persistence (t-1)	**0.04826**	0.03847	LightGBM	0.22002	0.20040

Note: RMSE and MAE are calculated over the 2022–2023 holdout test period. Bold values denote the best performance in terms of the RMSE. Additional evaluation metrics, rolling-origin evaluation results, 95% bootstrap confidence intervals, and residual autocorrelation diagnostics are provided in S2 Appendix B (Tables B1–B4).

Specifically, for the total HIE, the persistence model recorded an RMSE of 0.09297, significantly outperforming the best nonlinear indicator-based ensemble model, LightGBM (RMSE = 0.24231). A similar pattern is observed for medical institution expenditure, where the persistence model yielded an RMSE of 0.11088, whereas the best nonlinear ensemble comparator, gradient boosting, recorded an RMSE of 0.19807. The contrast was most pronounced for pharmacy expenditure, where the persistence model achieved an RMSE of 0.04826, whereas LightGBM recorded an RMSE of 0.22002, among the nonlinear indicator-based ensemble models. These results indicate that the simple univariate autoregressive baselines remain highly competitive for the short-term forecasting of the national HIE. However, such models do not incorporate national statistical indicators; therefore, they cannot support policy-relevant explanatory analysis. Consequently, regularized linear and nonlinear indicator-based models were examined as candidates for SHAP-based explanatory analyses.

### Model selection for a SHAP-based explanatory analysis

Although forecasting accuracy is essential, the primary objective of this study is to identify and interpret the model-conditional contributions of national economic, social, and financial indicators to national HIE predictions. From this perspective, univariate baseline models—despite their strong predictive performance—are unsuitable for a SHAP-based explanatory analysis because they do not incorporate national statistical indicators, and cannot quantify the contribution of external factors.

Accordingly, model selection for explanatory analyses is based on four criteria: (1) the ability to incorporate national statistical indicators, (2) competitive predictive performance across all expenditure components, (3) computational efficiency and reproducibility, and (4) compatibility with SHAP-based interpretability.

Table 8 compares the regularized linear and nonlinear indicator-based candidate models using both the test-period and rolling-origin RMSE, along with their interpretability and analytical roles. The results indicate that the regularized linear indicator-based benchmarks, including Ridge, LASSO, and ElasticNet, achieved comparatively low RMSE values across all the expenditure components, thus suggesting a significant model-conditional linear predictive relevance for the national HIE. In particular, LASSO and ElasticNet demonstrate strong predictive accuracy for the total and pharmacy HIEs, with RMSE values below 0.12 and 0.10, respectively.

**Table 8:** Comparison of the regularized linear and nonlinear indicator-based candidate models using test and rolling-origin RMSE values and analytical roles.

Model	Total		Medical institutions		Pharmacies		Interpretability	Analytical role
	Test	ROE	Test	ROE	Test	ROE		
Ridge regression	0.12457	0.05504	0.13686	0.06541	0.10862	0.05338	Coefficient-based linear	Linear predictive benchmark under L2 regularization
LASSO regression	0.10981	0.04973	0.12495	0.06080	0.09945	0.04632	Sparse coefficient-based linear	Sparse linear predictive benchmark
ElasticNet	0.11346	0.05082	0.12753	0.06107	0.09100	0.04687	Regularized coefficient-based linear	Primary linear robustness comparator for feature-importance sensitivity analysis
RF	0.25649	0.04838	0.25626	0.05664	0.27515	0.04330	Tree-based SHAP-compatible	Bagging-based nonlinear robustness benchmark
Gradient boosting	0.27950	0.04955	0.19807	0.06664	0.28412	0.04957	Tree-based SHAP-compatible	Boosting-based nonlinear benchmark with category-specific predictive strength
XGBoost	0.26334	0.05169	0.24204	0.07921	0.36235	0.04897	Tree-based SHAP-compatible	Regularized boosting benchmark for nonlinear comparison
LightGBM	0.24231	0.04778	0.23934	0.05913	0.22002	0.04897	Tree-based SHAP-compatible	Primary nonlinear explanatory model for SHAP analysis

Note: The test-period RMSE values were calculated for the 2022–2023 holdout test period, whereas the ROE mean RMSE values were calculated using rolling-origin evaluation with an expanding window. The univariate baseline models are excluded because they do not incorporate national statistical indicators, which renders them unsuitable for SHAP-based explanatory analyses. The models serve distinct analytical roles: the linear models (i.e., Ridge, LASSO, and ElasticNet) function as predictive benchmarks, with ElasticNet additionally serving as a robustness comparator for the feature-importance sensitivity analysis; LightGBM is the primary nonlinear explanatory model owing to its balanced cross-category ROE performance, SHAP compatibility, and capacity to represent nonlinear macrofinancial associations. The model selection for the explanatory analysis is not determined by the test-period RMSE ranking alone. ROE = rolling-origin evaluation. Additional performance metrics, confidence intervals, and residual diagnostics are reported in S2 Appendix B (Tables B1–B4).

In this study, LightGBM was not selected as the best forecasting model. Instead, it was used as the primary nonlinear explanatory model for SHAP-based interpretation, despite the lower test-period RMSE achieved by linear models, owing to three considerations.

First, LASSO and ElasticNet provide strong predictive benchmarks, but their standard linear specifications impose additive relationships and cannot readily represent nonlinear, stepwise, plateau-like, or interaction patterns without additional feature engineering. The SHAP dependence plots presented below depict stepwise, plateau-like, and—in some cases—threshold-like response patterns, suggesting that a nonlinear explanatory model is useful for interpreting the macrofinancial associations with HIE.

Second, tree-based SHAP explanations may be sensitive under multicollinearity and distribute importance across correlated predictors. Therefore, the LightGBM-SHAP results are not interpreted as definitive evidence for variable selection. We compared the mean absolute SHAP rankings for LightGBM with the ElasticNet-based standardized absolute coefficient rankings to assess the model dependence. This sensitivity analysis showed a limited agreement, indicating that the individual feature rankings vary across nonlinear and regularized linear specifications. Therefore, the SHAP rankings are interpreted as model-conditional explanatory signals rather than as robust standalone variable-selection results. The quantitative multicollinearity diagnostics supported this interpretation. Among the 1,770 pairwise Pearson correlations calculated from the 60 national statistical indicators, 675 pairs had |r| ≥ 0.70 and 277 pairs had |r| ≥ 0.90, with the highest correlation observed between aoi15 and iai52 (r = 0.9999). VIF diagnostics also indicated an extensive linear dependence among the indicators: all 60 indicators exceeded the VIF threshold of 5, and 52 indicators exceeded the VIF threshold of 10, with the highest VIF observed for eai51 (VIF = 10,897,589.74). These findings indicate that the national indicators contain correlated macrofinancial clusters. Accordingly, the individual SHAP rankings should be interpreted as model-conditional and potentially cluster-level monitoring signals rather than independent variable-specific effects. The full pairwise correlation and VIF diagnostics are reported in S3 Appendix C, Tables C6 and C7.

Third, under ROE, which more closely reflects the operational forecasting, LightGBM achieves a competitive performance relative to the linear models, particularly for the total HIE. For example, the ROE RMSE of LightGBM for the total HIE was 0.04778, which was comparable to or slightly better than that of LASSO and ElasticNet. This suggests that the test-period RMSE gap should be interpreted considering the accuracy–interpretability tradeoff rather than as a systematic explanatory inferiority. Therefore, LightGBM was used as a unified nonlinear explanatory model, whereas Ridge, LASSO, and ElasticNet were retained as regularized linear predictive benchmarks and model-dependence comparators.

### SHAP-based importance of national indicators

The SHAP analysis was conducted using the selected LightGBM model to quantify the relative contribution of the explanatory variables to the national HIE. In addition to the 60 national statistical indicators, lagged expenditure terms were included as control variables to capture the strong autoregressive structure of the monthly national HIE. This design enabled the model to separate the persistence effects from the incremental explanatory role of the national indicators.

Fig 3 presents the top eight features ranked by the mean absolute SHAP values for each expenditure component. Across all three series, the lagged expenditure variables appear to be among the highest-ranked features, confirming the dominant role of temporal persistence in the national HIE. This result is consistent with the aforementioned overall forecasting-performance results and supports including the autoregressive controls in the explanatory model.

**Fig 3:**
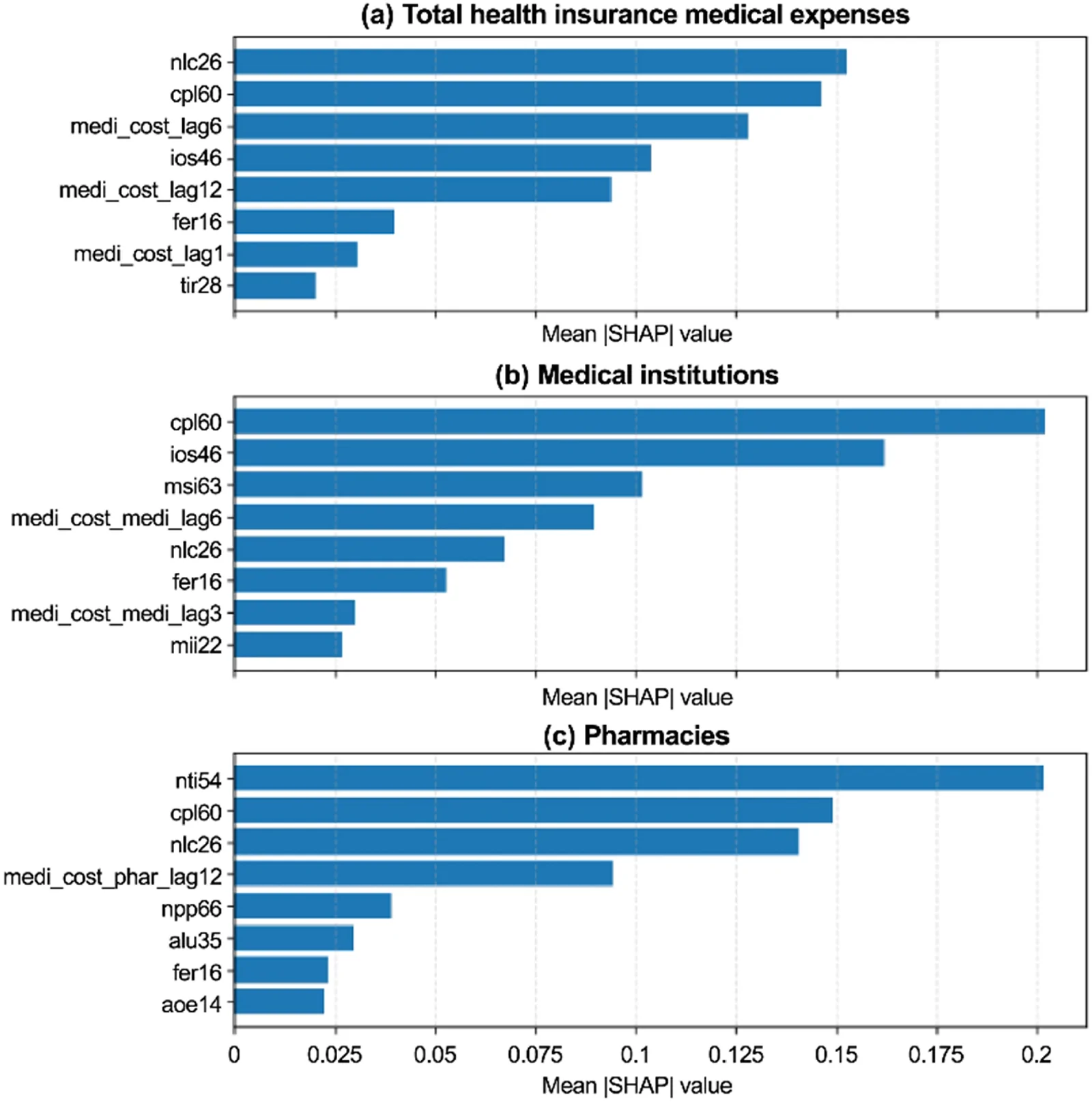
Top eight features ranked by mean absolute SHAP value under the lag-controlled LightGBM models for each HIE category.

Notably, even after accounting for strong persistence effects, several national statistical indicators remain highly ranked, indicating that the macroeconomic, social, and financial conditions provide an additional explanatory power beyond a simple time dependence. Given the presence of correlated macrofinancial indicators, the SHAP importance scores should be interpreted as cluster-level signals of macrofinancial relevance rather than precise estimates of the importance of individual variables. Indicators sharing economic characteristics—such as price-level proxies or activity-scale measures—may jointly contribute to the expenditure dynamics in ways that SHAP partially distributes across the cluster, implying that the policy relevance of identified indicators should be assessed at the conceptual category level rather than for each variable in isolation. Among these, the cost-of-living index (cpl60) consistently ranks among the top national indicators across all expenditure components, suggesting that price-level dynamics are associated with national HIE predictions after accounting for past expenditure levels.

Simultaneously, clear category-specific patterns emerge when the analysis focuses on the national indicators rather than the lagged terms. For the total HIE, indicators reflecting the overall economic scale and service-sector activity—such as the number of listed companies (nlc26) and the index of services (ios46)—rank prominently, suggesting that the aggregate HIE predictions are associated with broad macroeconomic conditions. For the medical institution expenditure, consumption- and price-related indicators, including cpl60 and the automobile sales index (msi63), show relatively stronger model-conditional contributions, whereas the pharmacy expenditure predictions are more strongly associated with external indicators of economic conditions, as reflected by the prominence of the net goods terms of trade index (nti54).

Table 9 summarizes the national indicators that overlap across categories as well as those that are uniquely important for medical institutions and pharmacies to highlight the distinction between common and provider-specific monitoring indicators, with the lagged control variables excluded from interpretation. The results highlight cpl60 and nlc26 as crosscutting monitoring indicators, whereas msi63 is more specific to medical institutions, and nti54 and npp66 (i.e., the number of people moving in the population) are more specific to the pharmacy expenditure.

**Table 9:** Overlapping and category-specific SHAP indicators (top eight indicators).

Category	Macroeconomic variables	Key indicators	Description
Common top indicators	Growth	nlc26	Number of listed companies
	Prices	cpl60	Cost-of-living index
	Global	fer16	Foreign exchange reserves
Indicators specific to medical institutions	Prices	msi63	Automobile sales index
	Production	mii22	Manufacturing inventory index
Indicators specific to pharmacies	Global	nti54	Net goods terms of trade index
	Population	npp66	Number of people moving in population
	Raw materials	alu35	Aluminum

Finally, Table 10 reports the top three SHAP-ranked national indicators for each HIE category, providing a parsimonious set of variables for policy-relevant interpretation. By conditioning on the lagged HIE, these rankings reflect the incremental importance of the national statistical indicators, thereby offering a more policy-informative view of the category-specific monitoring indicators underlying the HIE dynamics.

**Table 10:** Top three category-specific SHAP indicators.

Category	Ranking	Macroeconomic variables	Feature	Description
Total HIE	1	Growth	nlc26	Number of listed companies
	2	Prices	cpl60	Cost-of-living index
	3	Growth	ios46	Index of services
Medical institution HIE	1	Prices	cpl60	Cost-of-living index
	2	Growth	ios46	Index of services
	3	Prices	msi63	Automobile sales index
Pharmacy HIE	1	Global	nti54	Net goods terms of trade index
	2	Prices	cpl60	Cost-of-living index
	3	Growth	nlc26	Number of listed companies

Only the top-ranked national indicators and their aggregate importance are discussed here to enhance interpretability, and respect journal space constraints. The directional effects and nonlinear relationships for the most policy-relevant variables are selectively illustrated, whereas the complete top 20 SHAP feature rankings, including the lagged expenditure control terms and national statistical indicators, are reported in S3 Appendix C (Figs C1–C3).

Fig 4 shows the SHAP dependence plots for selected national statistical indicators derived from the category-specific LightGBM explanatory models. These plots illustrate the contribution of each indicator to the national HIE after controlling for strong autoregressive effects using lagged HIE terms. They illustrate the stepwise, plateau-like, and threshold-like model-estimated contribution patterns of key macroeconomic indicators in the HIE predictions, rather than emphasizing their short-term volatility.

**Fig 4:**
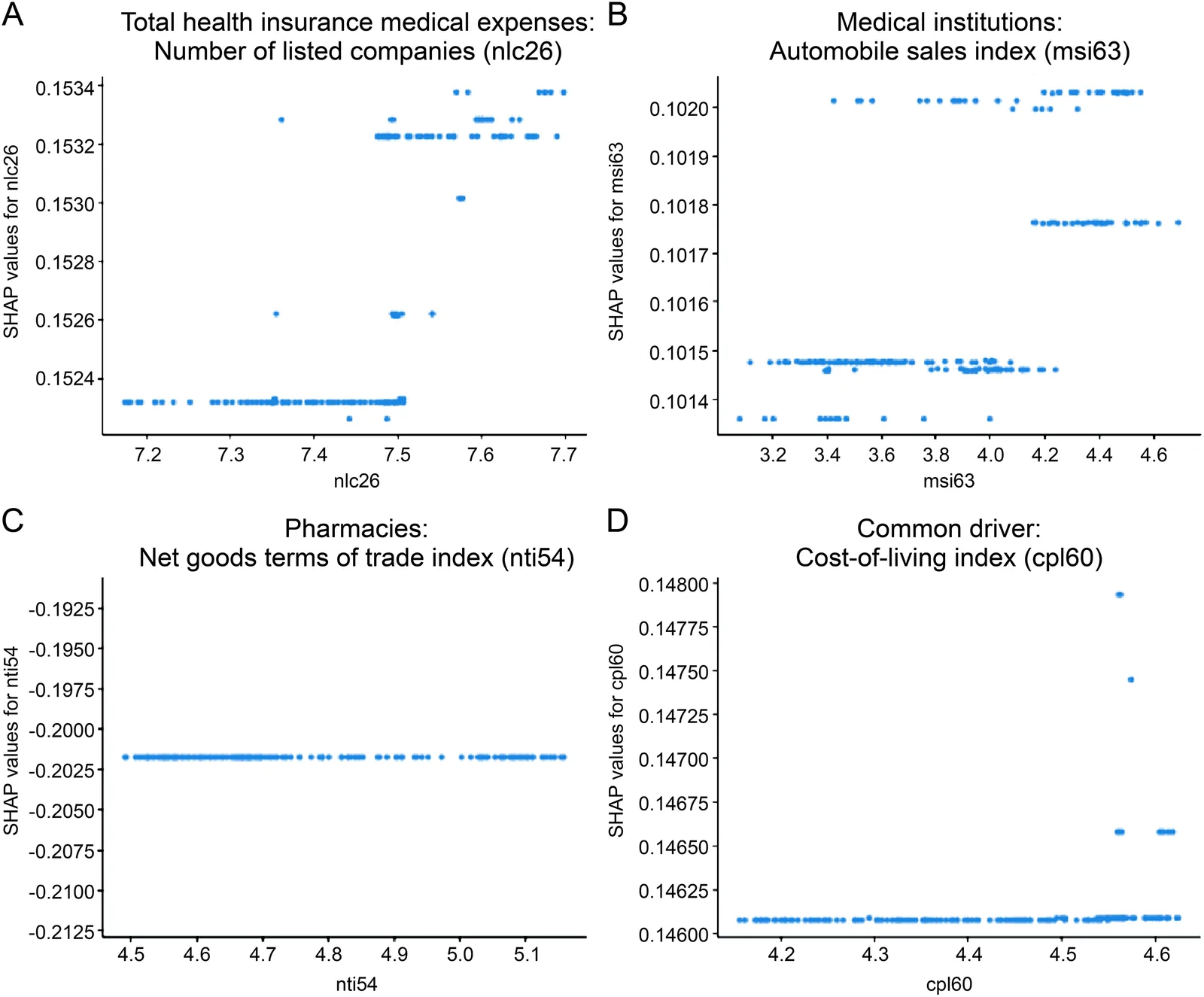
SHAP dependence plots for selected national statistical indicators derived from category-specific, lag-controlled LightGBM explanatory models. Note: Panels A–C are generated from separately optimized LightGBM models corresponding to different dependent variables: Panel A, total national HIE; Panel B, medical institution HIE; and Panel C, pharmacy HIE. Panel D shows the dependence plot for a common macro-financial indicator using the total HIE model as a representative case. In all models, lagged expenditure terms are included as control variables to account for strong temporal persistence but are omitted from the visualization to highlight the model-estimated contributions of national statistical indicators.

Panel A shows that the number of listed companies (nlc26), which represents the overall economic scale, has a threshold-type relationship with the total national HIE. The stepwise pattern reflects the tree-based structure of the model, and suggests that economic expansion is associated with discrete increases in the expenditure contributions beyond certain levels. Panel B indicates that the automobile sales index (msi63), a proxy for domestic consumption conditions, plays a differentiated role in the HIEs associated with medical institutions, with distinct contribution levels across value ranges. This supports the interpretation that the predictions of the HIEs of medical institutions show stronger model-estimated associations with internal demand-side conditions.

Panel C presents the dependence plot for the net goods terms of the trade index (nti54) under the pharmacy HIE model. The relatively flat yet consistently negative SHAP values indicate that external economic conditions are associated with relatively stable negative model contributions in the pharmacy HIE predictions rather than large marginal fluctuations. This pattern suggests that trade-related conditions may represent the background macrofinancial conditions associated with the pharmacy HIE predictions. Finally, Panel D shows the plot for the cost-of-living index (cpl60), a common monitoring indicator across the HIE components. The predominantly flat pattern, with localized deviations at higher values, suggests that price-level changes function as persistent macroeconomic pressure signals rather than short-term marginal drivers in the fitted model.

Overall, Fig 4 complements the SHAP importance rankings by showing that the selected high-ranking national indicators are associated with the HIE predictions through stepwise, plateau-like, and threshold-like SHAP response patterns within the fitted LightGBM models, rather than through simple linear gradients. These findings support the interpretation that the national HIE predictions are conditionally associated with the broader macrofinancial conditions, and underscore the policy relevance of incorporating macroeconomic indicators into system-level expenditure monitoring.

As the SHAP results in Tables 9 and 10 represent the model-conditional predictive importance rather than causal evidence, the top-ranked indicators were subsequently subjected to a Granger-type temporal predictive validation. This additional analysis examined whether the SHAP-identified indicators showed a statistically supported exploratory lagged monitoring relevance beyond expenditure persistence after stationarity transformation and autoregressive controls.

### Temporal predictive validation results

Table 11 presents the Granger-type temporal predictive validation results for the SHAP-selected indicators across three expenditure categories: total, medical institution, and pharmacy HIEs. For each indicator, the table reports the selected lag yielding the strongest lagged monitoring signal within the specified 1–12-month window.

**Table 11:** Granger-type temporal predictive validation of the SHAP-selected indicators using stationarity-transformed series.

Category	Indicator	Selected Lag (months)	Test Statistic (F-score)	p-value	FDR-adjusted p-value	Bonferroni-adjusted p-value	Exploratory Lagged Support	Interpretation
Total HIE	ios46	12	4.5525	1.20E-06	6.00E-06	6.00e-06	Yes	Bonferroni-supported lagged monitoring evidence
	cpl60	7	4.2294	0.0002	0.0005	0.0010	Yes	Bonferroni-supported lagged monitoring evidence
	nlc26	1	7.2550	0.0075	0.0125	0.0375	Yes	Bonferroni-supported lagged monitoring evidence
	tir28	1	2.0283	0.1555	0.1944	0.7774	No	Not statistically supported
	fer16	5	1.4839	0.1952	0.1952	0.9760	No	Not statistically supported
Medical institution HIE	cpl60	1	17.5433	3.76E-05	0.0001	0.0002	Yes	Bonferroni-supported lagged monitoring evidence
	ios46	10	3.9050	5.88E-05	0.0001	0.0003	Yes	Bonferroni-supported lagged monitoring evidence
	nlc26	1	6.4411	0.0117	0.0195	0.0584	Yes	FDR-supported exploratory lagged monitoring evidence
	msi63	2	2.9282	0.0551	0.0689	0.2757	No	Not statistically supported
	fer16	5	0.6154	0.6882	0.6882	1.0000	No	Not statistically supported
Pharmacy HIE	cpl60	11	5.8673	1.77E-08	8.85E-08	8.85E-08	Yes	Bonferroni-supported lagged monitoring evidence
	npp66	11	4.4714	3.61E-06	9.02E-06	1.80E-05	Yes	Bonferroni-supported lagged monitoring evidence
	nlc26	12	3.6892	3.95E-05	6.58E-05	0.0002	Yes	Bonferroni-supported lagged monitoring evidence
	nti54	12	1.5130	0.1196	0.1495	0.5979	No	Not statistically supported
	alu35	9	0.9198	0.5083	0.5083	1.0000	No	Not statistically supported

Note: Granger-type tests were conducted only for the SHAP-selected indicators using stationarity-transformed series. Series with nonnegative values were transformed using first-order log1p differencing, whereas variables unsuitable for logarithmic differencing were transformed using first differences. Some transformed indicators remained mixed or inconclusive, according to the combined ADF and KPSS diagnostics; however, the results involving these indicators were retained for supplementary temporal screening and interpreted cautiously given the incomplete stationarity support in the transformed series, rather than as fully robust temporal predictive evidence. The selected lag indicates the lag between 1 and 12 months that produced the smallest SSR_F-test p-value for each SHAP-selected indicator within the specified exploratory lag window. The FDR- and Bonferroni-adjusted p-values reported in this table were calculated within the SHAP-selected test family for each expenditure category and do not by themselves correct for the lag-search procedure across the 12 candidate lags. Therefore, a supplementary lag-search-adjusted sensitivity analysis, using the Bonferroni correction across the 12 candidate lags before a category-level multiplicity correction, is reported in S2 Appendix B, Table B6. In the main temporal screening table, the indicators with FDR-adjusted p-values < 0.05 were classified as indicators with exploratory lagged monitoring support, whereas the results that survived the Bonferroni correction were interpreted as stronger statistical support within the SHAP-selected temporal screening family. However, these main-table classifications should be interpreted together with the lag-search-adjusted sensitivity results in S2 Appendix B, Table B6, and the residual autocorrelation diagnostics in S2 Appendix B, Table B5. Overall, the results should be interpreted as exploratory temporal monitoring signals rather than a confirmation of robust lagged predictive relationships or structural causal effects.

The main screening analysis of the items presented in Table 11 revealed that three SHAP-selected indicators for total HIE, namely ios46, cpl60, and nlc26, met the FDR and Bonferroni thresholds, with selected lags of 12, 7, and 1 months, respectively. However, the lag-search-adjusted sensitivity analysis presented in S2 Appendix B (Table B6) indicated that ios46 and cpl60 retained stronger support after additional lag-search adjustment, whereas nlc26 was attenuated and should be interpreted as a weaker post-hoc screening signal. By contrast, the 3-year treasury bond interest rate (tir28) and foreign exchange reserves (fer16) did not retain statistical support in either the main screening analysis or the lag-search-adjusted sensitivity analysis. These findings suggest that service-sector activity and price-level conditions provided more stable exploratory temporal monitoring signals for aggregate HIE dynamics, and economic-scale-related signals such as nlc26 should be interpreted more cautiously.

For medical institution HIE, the main temporal screening analysis of the items in Table 11 identified cpl60, ios46, and nlc26 as indicators with exploratory lagged monitoring support after multiple-testing correction. cpl60 and ios46 showed stronger Bonferroni-supported lagged monitoring evidence, whereas nlc26 retained FDR-supported exploratory support in the main temporal screening analysis. However, the lag-search-adjusted sensitivity analysis in S2 Appendix B (Table B6) indicated that cpl60 and ios46 retained stronger support after additional lag-search adjustment, whereas nlc26 was attenuated and should therefore be interpreted as a weaker post-hoc screening signal. By contrast, msi63, although relatively important in the SHAP ranking, and fer16 were not statistically supported in the lag-search-adjusted sensitivity analysis. This divergence reinforces the notion that SHAP-based importance should be interpreted as model-conditional explanatory relevance rather than as direct evidence of stable lagged temporal predictiveness.

For pharmacy HIE, the main screening analysis of the items in Table 11 was more selective than the SHAP rankings. cpl60, npp66, and nlc26 met the FDR and Bonferroni thresholds in the main screening analysis. The lag-search-adjusted sensitivity analysis in S2 Appendix B (Table B6) further indicated that cpl60, npp66, and nlc26 retained support after additional lag-search and category-level correction, suggesting relatively more stable exploratory monitoring support for pharmacy HIE. However, the nti54, despite being the highest-ranked SHAP indicator for pharmacy HIE, and aluminum prices (alu35) were not statistically supported in either the main screening analysis or the lag-search-adjusted sensitivity analysis. These results indicate that high SHAP importance does not necessarily translate into stable lagged monitoring support after stationarity transformation, autoregressive controls, multiple-testing correction, and lag-search adjustment.

Overall, the divergence between SHAP rankings and temporal screening classifications reflects their different analytical roles: SHAP summarizes model-conditional predictive importance, whereas Granger-type screening assesses whether lagged indicators provide additional exploratory monitoring information beyond autoregressive expenditure persistence.

To evaluate the sensitivity of these findings to the minimum-p-value lag-selection procedure, we additionally conducted a lag-search-adjusted analysis in which the selected minimum p-value for each indicator was conservatively adjusted across the 12 candidate lags before category-level FDR and Bonferroni correction. These results are reported in S2 Appendix B (Table B6). After this additional adjustment, ios46 and cpl60 for total HIE; cpl60 and ios46 for medical institution HIE; and cpl60, npp66, and nlc26 for pharmacy HIE retained support after lag-search and category-level correction and were therefore interpreted as more stable exploratory monitoring signals. By contrast, nlc26 for total HIE and medical institution HIE was attenuated after lag-search adjustment and was therefore treated as a weaker post-hoc screening signal. The remaining indicators, namely tir28 and fer16 for total HIE, msi63 and fer16 for medical institution HIE, and nti54 and alu35 for pharmacy HIE, were not statistically supported in the lag-search-adjusted sensitivity analysis. Overall, this sensitivity analysis further supports the interpretation of the Granger-type results as exploratory monitoring evidence rather than confirmatory temporal validation.

Residual autocorrelation diagnostics for the corresponding Granger-type lagged regression models are reported in S2 Appendix B (Table B5). The interpretation labels in Table 11 indicate statistical support after FDR and Bonferroni correction, rather than the absence of residual dependence; therefore, results with significant residual autocorrelation were interpreted cautiously as indicative temporal monitoring signals.

Fig 5 integrates the SHAP-ranked indicators with the main temporal screening results for the items in Table 11 across the three HIE components. Panel A shows the results for total HIE. nlc26, cpl60, and ios46 ranked highest in SHAP importance and showed exploratory lagged monitoring support in the main screening analysis, whereas fer16 and tir28 did not. However, the supplementary diagnostics further differentiated these signals: ios46 and cpl60 retained support after lag-search adjustment and showed no residual autocorrelation in the corresponding lagged regression models, whereas nlc26 had mixed or inconclusive stationarity diagnostics, showed residual autocorrelation, and was attenuated after lag-search adjustment. Thus, ios46 and cpl60 were the more diagnostically stable exploratory monitoring signals for total HIE.

**Fig 5:**
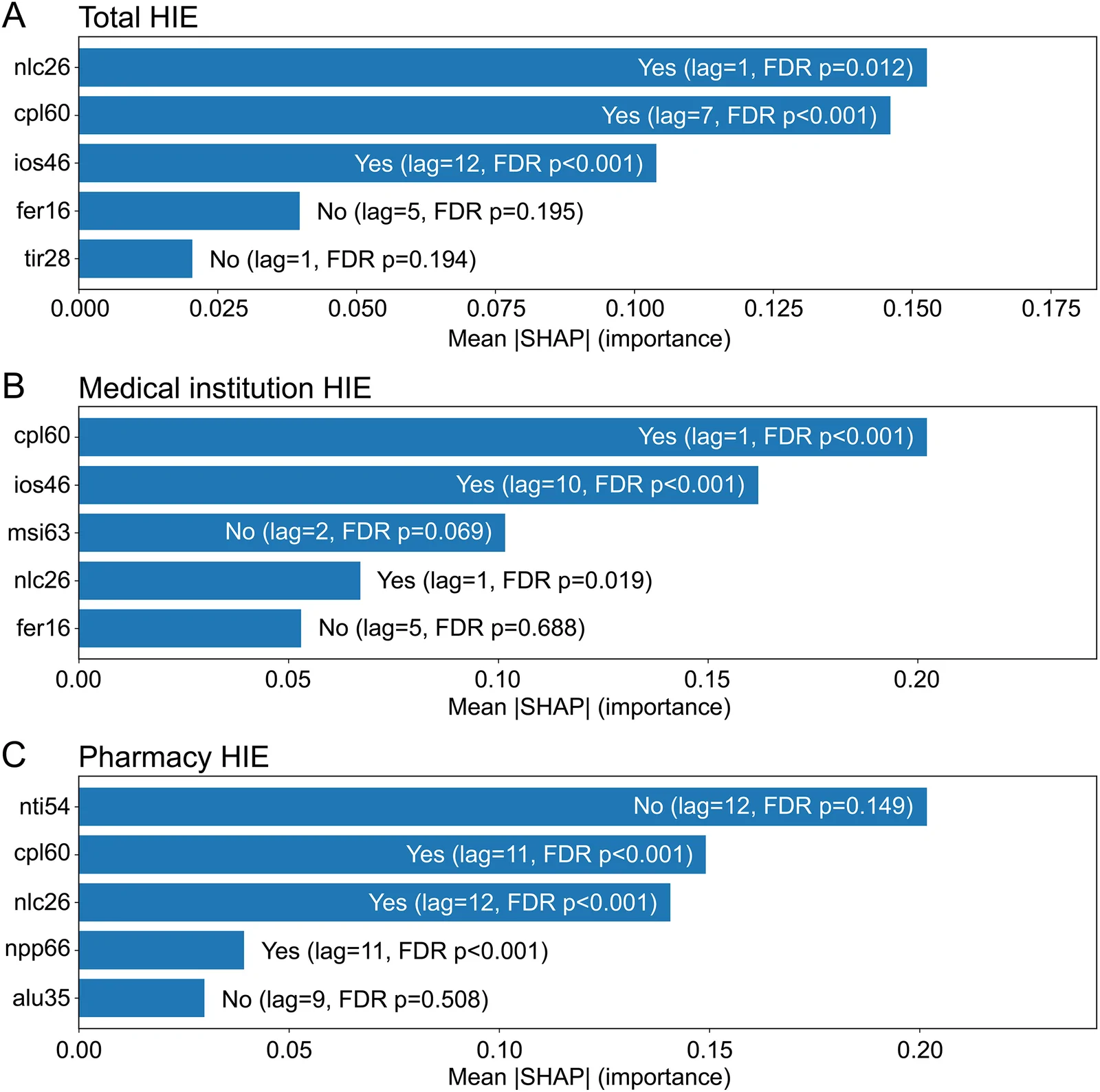
Integrated SHAP-ranked indicators and Granger-type temporal screening results across HIE components. Note: Panels A–C show total HIE, medical institution HIE, and pharmacy HIE, respectively. Bars represent mean absolute SHAP values from the selected LightGBM explanatory models, and annotations report the main Table 11 temporal screening classification, selected lag, and FDR-adjusted p-value. Lag-search-adjusted sensitivity results are reported in S2 Appendix B, Table B6.

Panel B shows the results for medical institution HIE. cpl60 and ios46 were highly ranked by SHAP and showed stronger Bonferroni-supported monitoring evidence in the main screening analysis, whereas nlc26 showed weaker FDR-supported evidence. msi63 and fer16 did not show exploratory lagged monitoring support. The lag-search-adjusted sensitivity analysis retained cpl60 and ios46 but attenuated nlc26. Residual autocorrelation in several medical institution HIE lagged regression models, together with the mixed or inconclusive stationarity result for nlc26, further requires cautious interpretation of these temporal screening results.

Panel C shows the results for pharmacy HIE. The contrast between SHAP importance and temporal screening was particularly evident for nti54, which was the highest-ranked SHAP indicator but did not show exploratory lagged monitoring support. By contrast, cpl60, npp66, and nlc26 showed support in the main screening analysis and retained support after lag-search and category-level correction, whereas alu35 did not show support. However, residual autocorrelation was detected across the pharmacy HIE lagged regression models, and nlc26 additionally had mixed or inconclusive stationarity diagnostics. Accordingly, the supported pharmacy indicators should be interpreted as diagnostically qualified, hypothesis-generating monitoring signals rather than as robust lagged predictive evidence.

Overall, Fig 5 visually demonstrates that SHAP-based predictive importance and exploratory temporal screening support only partially overlap across expenditure components. By presenting SHAP importance together with the main screening classifications, selected lags, and FDR-adjusted p-values of the items in Table 11, the figure provides a visual synthesis of the two evidence layers, and detailed statistical classifications and diagnostic qualifications are reported in Table 11, S2 Appendix B (Tables B5 and B6), and S4 Appendix D. The combined evidence therefore supports a smaller set of hypothesis-generating monitoring signals rather than evidence of stable structural transmission or causal effects.

### Summary of key findings

This study yielded five principal findings.

First, persistence-based baselines provided the strongest short-term forecasts, confirming the dominant autoregressive structure of monthly HIE.

Second, although indicator-based ML models did not outperform the strongest univariate baselines in short-term forecasting, they remained useful for monitoring-oriented explanatory interpretation because they incorporated national statistical indicators, allowing HIE variation to be examined in relation to external macroeconomic, social, and financial conditions.

Third, although regularized linear indicator-based models achieved lower test-period RMSE than LightGBM, LightGBM was used as the primary nonlinear explanatory model rather than as the best forecasting model. This choice supported a unified SHAP-based interpretation of nonlinear and category-specific predictive patterns across total, medical institution, and pharmacy HIE, while the regularized linear models remained predictive benchmarks and model-dependence comparators, with ElasticNet additionally serving as a robustness comparator.

Fourth, SHAP identified both shared macro-level and provider-specific predictive patterns across HIE components; these patterns should be interpreted as model-based predictive importance rather than causal effects.

Finally, only a subset of SHAP-selected indicators showed exploratory lagged monitoring support after stationarity transformation and multiple-testing correction, with residual autocorrelation diagnostics and the lag-search-adjusted sensitivity analysis further qualifying the strength of evidence.

Overall, these findings provide coherent evidence to link forecasting performance, model-based interpretability, and supplementary exploratory temporal monitoring evidence within a unified analytical framework. The results suggest that SHAP-selected macro-financial indicators may inform HIE risk-monitoring hypotheses and contextual review. However, they do not establish structural causal mechanisms, confirmed surveillance signals, or counterfactual policy effects.

## Discussion

The results require a clear distinction between model-based predictive importance and exploratory lagged monitoring support. As shown in Table 11 and Fig 5, only a subset of SHAP-ranked indicators showed exploratory support in the main temporal screening analysis, indicating that model importance should not be interpreted as equivalent to temporal monitoring evidence. SHAP-selected indicators are therefore treated as candidate monitoring signals, with stronger interpretation reserved for those also supported by temporal screening.

Because the analysis is based on national aggregate monthly data, these findings should be interpreted at the system-monitoring level rather than as evidence of individual-, institution-, regional-, or policy-level causation. They do not establish that changes in these indicators would causally alter HIE trajectories; aggregate co-movement may also reflect demographic change, institutional reforms, reimbursement policies, insurance coverage changes, or other unobserved system-level factors.

From a financial management standpoint, the findings indicate that national HIE is associated with broader macro-financial environments in addition to its strong autoregressive dynamics: even after controlling for expenditure persistence, indicators related to price levels, economic activity, and external conditions retained model-conditional relevance. Public insurance spending may therefore be examined as a macro-sensitive fiscal exposure, with the exploratory Granger-type temporal screening results treated as hypotheses for further fiscal monitoring review rather than as evidence of established structural or causal relationships.

Accordingly, the primary objective of this study is not to outperform univariate benchmarks in short-term prediction; forecasting serves as a methodological tool to assess whether national statistical indicators add explanatory value beyond the strong autoregressive structure of monthly HIE.

The practical value of the indicator-based framework therefore lies not in replacing the persistence model for short-term point forecasting but in adding interpretable monitoring information around the autoregressive forecast. Although a persistence forecast can indicate the expected continuation of recent expenditure levels, it cannot explain which external conditions are associated with elevated expenditure risk or whether such associations differ between medical institutions and pharmacies. By contrast, the proposed framework identifies macro-financial contexts, such as price-level conditions, economic activity, external economic conditions, and population-related signals, that are conditionally associated with HIE predictions after accounting for lagged expenditure persistence. It also distinguishes indicators that are important only within the SHAP model from those that additionally show statistically supported exploratory lagged monitoring relevance in the Granger-type temporal screening analysis. This distinction allows fiscal monitoring to use SHAP-ranked indicators with exploratory temporal screening support as hypothesis-generating monitoring candidates while treating SHAP-ranked indicators without such support as contextual variables for further risk review alongside persistence-based baseline forecasts. These uses should be interpreted as hypothesis-generating monitoring support rather than as automatic intervention rules, operational surveillance criteria, or causal policy triggers.

Therefore, the explanatory role of macro-financial indicators is evaluated in terms of their persistence-adjusted conditional contributions, SHAP-based importance patterns, and exploratory lagged monitoring support following stationarity transformation and multiple-testing correction, rather than based on their ability to outperform autoregressive benchmarks in short-term forecasting.

Consistent with this distinction, cpl60, nlc26, and ios46 combined high SHAP importance with exploratory lagged monitoring support in the main temporal screening analysis, whereas nti54, important in the pharmacy SHAP ranking, did not meet the FDR threshold. This contrast illustrates why SHAP-based explanatory relevance should not be equated with temporal monitoring support and why the Granger-type screening results are treated as supplementary, hypothesis-generating evidence rather than as evidence of structural causal effects.

Therefore, from the perspective of feature selection, the analysis should be understood as post-hoc importance screening rather than premodel variable elimination. Retaining the full set of 60 indicators preserved broad macro-financial coverage during model training. The SHAP–ElasticNet comparison also indicates that individual rankings are model-dependent in the presence of correlated macroeconomic indicators. Therefore, the selected indicators are interpreted as model-conditional monitoring signals rather than as a definitive reduced feature set.

Finally, the dependence-plot evidence (Fig 4) complements the ranking tables by illustrating that key indicators are associated with expenditure through stepwise, plateau-like, and threshold-like SHAP response patterns within the fitted category-specific LightGBM models, which were separately optimized for total, medical institution, and pharmacy HIEs. Together, the results indicate that expenditure monitoring and risk review should distinguish between expenditure components rather than assume a uniform pattern. Macro-financial indicators, particularly price-level indicators, may also inform fiscal monitoring review beyond traditional within-system expenditure controls, with external-condition indicators considered provider-specific contextual variables.

### Financial and fiscal policy implications of macroeconomic factors for HIE

The SHAP results indicate that national HIE exhibits strong autoregressive dynamics while being conditionally associated with macroeconomic indicators that reflect price levels and economic scale. In particular, cpl60 consistently ranks as a key model-conditional monitoring indicator across total, medical institution, and pharmacy expenditure, suggesting that price-level conditions represent a shared macro-level signal relevant across provider types. The exploratory Granger-type temporal screening results are consistent with this monitoring-oriented interpretation, suggesting that cpl60 may be considered a hypothesis-generating price-level monitoring candidate for fiscal planning review rather than evidence of a structural causal effect.

These findings suggest that fiscal monitoring of public HIE may benefit from considering broader macro-financial indicators, particularly price-level conditions and indicators of economic activity. Rather than implying that macroeconomic indicators should be considered direct policy levers, the results suggest that these variables may serve as contextual inputs for scenario planning, fiscal monitoring review, and medium-term risk review. In particular, cpl60 may be considered a hypothesis-generating price-level monitoring candidate across expenditure components, whereas nlc26 and ios46 may provide contextual information on broader economic scale and service-sector activity. Given that cpl60 showed exploratory lagged monitoring support across all three expenditure categories, persistent upward movements in this indicator may warrant further monitoring review rather than immediate policy action. Incorporating these indicators into scenario-based fiscal stress testing may help generate fiscal monitoring hypotheses about macro-financial environments associated with elevated expenditure risk and support discussions on fiscal buffers, expenditure growth monitoring, and forward-looking insurance finance planning.

### Provider-specific differences between medical institution and pharmacy HIE components and their monitoring implications

By disaggregating national HIE into medical institution and pharmacy components, this study identifies provider-specific monitoring patterns rather than direct expenditure-determination mechanisms. SHAP-based evidence suggests that medical institution HIE predictions are more strongly associated with domestic consumption and price-related indicators, including cpl60 and msi63, whereas pharmacy HIE predictions show stronger model-conditional associations with external-condition and population-related indicators, including nti54 and npp66. However, the main Granger-type temporal screening analysis narrowed these SHAP-based patterns to a smaller set of indicators with exploratory lagged monitoring support: cpl60, ios46, and nlc26 for medical institution HIE and cpl60, npp66, and nlc26 for pharmacy HIE. Therefore, provider-specific SHAP patterns, particularly those involving msi63 and nti54, which did not meet the FDR threshold for exploratory lagged monitoring support in the main temporal screening analysis, should be interpreted as contextual monitoring evidence rather than as direct evidence of structural transmission channels. This distinction may inform differentiated fiscal risk monitoring hypotheses across provider types rather than uniform causal policy prescriptions.

These provider-specific monitoring differences may reflect variation in cost structures, utilization patterns, and supply-chain exposure. However, the present aggregate analysis does not directly identify these mechanisms. Therefore, the mechanisms underlying the observed monitoring patterns should be examined in future studies using provider-level, claims-level, regional, or supply-chain-related data.

From a fiscal and insurance policy perspective, the identified indicators are best interpreted as monitoring signals rather than direct policy levers. Although indicators such as cpl60, ios46, nlc26, and npp66 may help policy analysts monitor macro-financial conditions associated with future HIE movements, the present analysis does not establish that changing these indicators would causally alter expenditure trajectories. Therefore, the practical value of the framework lies in informing exploratory early-warning review, risk monitoring hypotheses, and explanatory review of expenditure trends, rather than prescribing causal intervention targets. This distinction is particularly important for national level HIE analysis, where macroeconomic co-movement, demographic shifts, insurance coverage changes, institutional reforms, and reimbursement policy changes may jointly shape expenditure dynamics.

### Policy applicability and limitations of explainable ML for public health insurance finance

This study contributes by applying SHAP-based interpretability to identify policy-relevant expenditure monitoring indicators. By incorporating lagged dependent variables as controls, the analysis demonstrates that explainable ML models can assess the model-conditional incremental contribution of national indicators in a highly autoregressive time-series setting.

However, this study has several limitations. First, SHAP values are model-conditional contribution measures rather than causal estimates; therefore, selected indicators should be interpreted as monitoring signals rather than intervention effects. Second, the national monthly aggregate design precludes inference about individual-level, institution-level, patient-level, regional-level, or counterfactual policy effects and may be affected by aggregation bias and unobserved system-level confounders, including demographic change, coverage expansion, reimbursement reforms, drug pricing policies, and pandemic-related utilization changes. Third, Granger-type tests identify lagged predictive precedence rather than structural mechanisms. Common macroeconomic co-movement and multicollinearity among the 60 national statistical indicators, as reflected in the pairwise correlation and VIF diagnostics reported in S3 Appendix C (Tables C6 and C7) may affect SHAP attribution and temporal validation results. Additionally, lag-selection uncertainty and strong autoregressive persistence may affect the Granger-type findings, and FDR correction does not eliminate the possibility of spurious associations. The lag-search-adjusted sensitivity analysis further showed that some indicators retained support after additional correction for the exploratory lag search, whereas others were attenuated, reinforcing the interpretation of the Granger-type results as hypothesis-generating monitoring evidence rather than confirmatory temporal validation. Residual autocorrelation diagnostics reported in S2 Appendix B further indicated that some temporal dependence remained in several lagged regression models, supporting cautious interpretation of the temporal validation results as indicative monitoring evidence rather than robust structural evidence. Fourth, the external validity of the findings is constrained by the institutional context of the Korean National Health Insurance system. Because all expenditure outcomes were measured from HIRA monthly reimbursement statistics, the observed macro-financial monitoring signals may reflect Korea-specific features of national reimbursement, benefit coverage, fee schedules, pharmaceutical pricing, and utilization management. Therefore, the identified associations should not be assumed to generalize directly to healthcare systems organized around private insurance, multi-payer arrangements, tax-financed National Health Service models, or reimbursement designs based on capitation, diagnosis-related groups, fee-for-service, or mixed payment systems. Cross-national replication and validation using data from healthcare systems with different insurance designs, reimbursement mechanisms, and policy-change periods are needed before these indicators can be interpreted as broadly generalizable monitoring signals. Future work using disaggregated claims, provider-level panels, regional data, explicit policy variables, or quasi-experimental designs is needed to evaluate mechanisms and heterogeneous effects, and cross-national validation remains necessary to establish the generalizability of the identified monitoring indicators.

Despite these limitations, the framework illustrates how explainable ML can serve as a practical bridge between prediction, explanation, and fiscal risk-monitoring hypothesis generation in public health insurance systems. Operationally, the framework may inform an indicator-based expenditure review system. Indicators that exhibit both high predictive contribution and exploratory lagged statistical support may be considered hypothesis-generating monitoring candidates, whereas those with weaker temporal support may serve as contextual variables for further review. Embedding these candidate indicators into a rolling monitoring dashboard with periodic model updates and diagnostic checks may help detect changes in predictive relationships and identify unstable or potentially spurious associations. However, any operational use as surveillance inputs requires external validation in independent datasets and institutional contexts.

### Interpretation of macro-financial monitoring signals and temporal predictive sensitivity

In the main screening analysis of the items in Table 11, cpl60, ios46, and nlc26 showed exploratory lagged monitoring support for both total and medical institution HIE, whereas cpl60, npp66, and nlc26 did so for pharmacy HIE. The lag-search-adjusted sensitivity analysis further qualified these results: ios46 and cpl60 for total HIE, cpl60 and ios46 for medical institution HIE, and cpl60, npp66, and nlc26 for pharmacy HIE retained stronger exploratory monitoring support, whereas nlc26 for total and medical institution HIE was attenuated after lag-search adjustment. These findings refine, rather than overturn, the main screening results. Accordingly, SHAP-ranked indicators remain model-conditional screening signals. Among them, those retaining exploratory temporal support may be considered hypothesis-generating monitoring candidates, whereas those with weaker or attenuated temporal support may serve as contextual variables for further review. The latter may reflect correlated macro-financial environments or proxy relationships rather than distinct or robust lagged predictive relationships.

The variation in selected lags suggests that expenditure monitoring may consider multiple time horizons rather than assuming a single uniform response pattern. In this regard, indicators with short-lag support may be considered for near-term exploratory review, whereas those with longer-lag patterns may inform broader medium-term monitoring hypotheses. Price-level conditions provide the most consistent cross-component monitoring context, whereas economic-activity, external-condition, and population-related indicators provide more component-specific contextual information. Although these patterns may inform exploratory early-warning review and forward-looking risk assessment, they should be interpreted as system-level, hypothesis-generating monitoring evidence from national aggregate time-series data rather than as intervention timing rules, operational surveillance criteria, or structural causal effects.

## Conclusions

This study develops an explainable ML framework with temporal predictive validation to analyze national HIE in relation to macroeconomic conditions. Although persistence-based baselines provide the strongest short-term forecasting performance, indicator-based models and SHAP analysis identify macro-financial variables that are conditionally associated with expenditure predictions across total, medical institution, and pharmacy HIE. The findings therefore support examining national HIE as a macro-sensitive fiscal risk exposure rather than solely as a healthcare-related expenditure trend.

Temporal predictive validation further showed that only a subset of SHAP-identified indicators provided exploratory lagged monitoring support, underscoring the distinction between predictive correlates and candidate monitoring indicators. Because the analysis is based on national aggregate time-series data, these results should be interpreted as system-level monitoring evidence rather than as individual-level, institution-level, regional-level, or counterfactual policy effects.

The empirical findings indicate that national HIE is predictively associated with broader macro-financial environments in addition to internal utilization dynamics. Price-level indicators may serve as cross-component, hypothesis-generating monitoring candidates, whereas provider-specific indicators reflect heterogeneous, model-conditional predictive patterns. Methodologically, the framework integrates predictive modeling, explainable AI, and temporal predictive validation to improve analytical rigor and interpretability and connect data-driven forecasting with policy-oriented monitoring.

From a policy standpoint, the framework may inform hypotheses for expenditure-risk monitoring and differentiated risk review across provider types. Indicators with both high predictive contribution and exploratory temporal support may be considered hypothesis-generating monitoring candidates, whereas high-importance indicators with weaker temporal support may serve as contextual variables for further review. Any operational use as a surveillance input requires external validation in independent datasets and institutional contexts. Because the identified indicators reflect the institutional context of the Korean National Health Insurance system, external validation is particularly important before application to healthcare systems with different reimbursement or insurance structures.

The temporal predictive validation used here relies on time-series statistical testing rather than on structural causal identification. Future research may strengthen causal inference using quasi-experimental designs, structural causal models, or more granular data. Overall, the framework may support fiscal risk monitoring and exploratory early-warning review while maintaining a clear distinction among prediction, monitoring evidence, and causal inference.

## Supporting information

S1 Appendix A. (DOCX)

S2 Appendix B. (DOCX)

S3 Appendix C. (DOCX)

S4 Appendix D. (DOCX)
