# Supplementary material for: Explainable machine learning framework with Granger-based temporal predictive validation for monitoring fiscal risk in national health insurance expenditure
Source: PLoS One. 2026 Oct 29;21(10):e0361904. doi: 10.1371/journal.pone.0361904 (PMC13719042; doi:10.1371/journal.pone.0361904)
Supplement: Supplementary file 1 [file pone.0361904.s001.docx]

**S1 Appendix A**

This appendix provides the complete descriptive statistics for the 60 national statistical indicators used in the analysis. The indicators are grouped by category and include minimum, maximum, mean, and standard deviation values.

Measurement units for all variables are reported in Table A1. Monetary variables are expressed in Korean won (KRW) or U.S. dollars (USD), population variables in thousand persons, and rate variables as percentages (%). Exchange rates are quoted as KRW per USD. Commodity prices follow standard international quotation units (e.g., USD per metric ton, USD per barrel, and USD per troy ounce). Index variables are presented according to their official definitions, including base-year normalized indices and standardized composite indices.

**Table A1. Descriptive statistics by category for the 60 national statistical indicators**

| Category | Feature | Description | Unit | Min | Max | Mean | Std.  Dev. |
| --- | --- | --- | --- | --- | --- | --- | --- |
| Employment | eer1 | Elderly employment rate | % | 51.3 | 71.1 | 63.028 | 4.24 |
|  | eur2 | Elderly unemployment rate | % | 1.2 | 5.4 | 2.49 | 0.59 |
|  | eap3 | Economically active population | thousand persons | 21442 | 29623 | 25723.94 | 2177.23 |
|  | noe4 | Number of employed | thousand persons | 20210 | 28835 | 24817.29 | 2146.60 |
|  | wer5 | Employment rate | % | 59.5 | 69.9 | 64.86 | 2.01 |
|  | wur6 | Unemployment rate | % | 2 | 5.7 | 3.53 | 0.59 |
|  | yea7 | Youth economically active population | thousand persons | 3974 | 5454 | 4498.53 | 396.57 |
|  | yur8 | Youth unemployment rate | % | 4.5 | 12.5 | 8.05 | 1.38 |
|  | yer9 | Youth employment rate | % | 38.5 | 47.8 | 42.87 | 2.14 |
| Global | aoe14 | Value of exports | 100 million USD | 110 | 638 | 368.67 | 139.51 |
|  | aoi15 | Value of imports | 100 million USD | 105 | 660 | 342.70 | 135.75 |
|  | fer16 | Foreign exchange reserves | 100 million USD | 768 | 4692 | 2925.99 | 1129.50 |
|  | wer17 | Won-dollar exchange rate | KRW per USD | 900.7 | 1534 | 1142.97 | 108.85 |
|  | eai51 | Export amount index | Index (2020=100) | 24.82 | 152.14 | 84.26 | 32.61 |
|  | iai52 | Import amount index | Index (2020=100) | 26.89 | 170.77 | 88.02 | 34.98 |
|  | itt53 | Income terms of trade index | Index (2020=100) | 35.74 | 116.94 | 74.96 | 21.82 |
|  | nti54 | Net goods terms of trade index | Index (2020=100) | 86.82 | 187.18 | 116.54 | 27.95 |
|  | ivi55 | Import volume index | Index (2020=100) | 38.72 | 122.76 | 77.86 | 21.63 |
|  | evi56 | Export volume index | Index (2020=100) | 19.86 | 119.92 | 70.52 | 29.58 |
| Interest rate | tir28 | 3-year treasury bond interest rate | % | 0.83 | 9.28 | 3.57 | 1.76 |
|  | tir29 | 5-year treasury bond interest rate | % | 1.09 | 10.02 | 3.81 | 1.82 |
|  | crr30 | Call rate | % | 0.48 | 5.37 | 2.83 | 1.38 |
|  | brr31 | Base rate | % | 0.5 | 5.25 | 2.83 | 1.35 |
|  | lir47 | Lending interest rate | % | 2.63 | 8.79 | 5.17 | 1.55 |
|  | rer48 | Receiving rate | % | 0.81 | 7.24 | 3.31 | 1.57 |
| Production | iip19 | Index of all industry production | Index (2020=100) | 48.3 | 126.5 | 84.58 | 17.65 |
|  | mpi20 | Manufacturing production index | Index (2020=100) | 58.4 | 117.1 | 90.42 | 13.55 |
|  | msi21 | Manufacturing shipments index | Index (2020=100) | 55.5 | 117.3 | 89.99 | 13.80 |
|  | mii22 | Manufacturing inventory index | Index (2020=100) | 55.7 | 124.5 | 88.55 | 18.51 |
|  | opo23 | Overseas plant orders | million USD | 51 | 64759 | 9769.96 | 12300.88 |
| Raw materials | alu35 | Aluminum | USD per metric ton | 1283 | 5216 | 1988.37 | 487.14 |
|  | lea36 | Lead | USD per metric ton | 412 | 3720 | 1701.01 | 720.41 |
|  | zin37 | Zinc | USD per metric ton | 748 | 4405 | 2094.56 | 845.74 |
|  | tin38 | Tin | USD per metric ton | 3695 | 44249 | 16749.24 | 8593.10 |
|  | nic39 | Nickel | USD per metric ton | 4828 | 52179 | 16222.04 | 7709.12 |
|  | iop43 | International oil price in Dubai | USD per barrel | 17.5 | 130.08 | 63.71 | 28.50 |
|  | gol44 | Gold | USD per troy ounce | 260.33 | 2034.7 | 1083.48 | 541.50 |
| Growth | ccc24 | Cyclical component of coincident index | Index (2020=100) | 96.4 | 102.6 | 100.08 | 1.067 |
|  | ccl25 | Cyclical component of leading index | Index (2020=100) | 96.6 | 105.2 | 100.26 | 1.45 |
|  | nlc26 | Number of listed companies | count | 1187 | 2541 | 1840.17 | 309.93 |
|  | mcs27 | Market capitalization | trillion KRW | 209.1 | 2747 | 1204.29 | 679.11 |
|  | bdr32 | Bill default rate | % | 0 | 0.63 | 0.04 | 0.07 |
|  | abd33 | Amount of bill dishonored | trillion KRW | 0 | 5.2 | 0.54 | 0.56 |
|  | nbd34 | Number of bill defaulters | count | 7 | 684 | 168.06 | 159.55 |
|  | kos40 | KOSPI Index | Index (1980.01.04 = 100) | 479.68 | 3296.68 | 1725.86 | 674.48 |
|  | kod41 | KOSDAQ Index | Index (1996.07.01 = 1,000) | 307.48 | 2663.7 | 662.68 | 250.21 |
|  | mcu42 | Manufacturing average capacity utilization rate | % | 62.7 | 83.8 | 76.43 | 3.63 |
|  | wri45 | Wholesale and retail index | Index (2020=100) | 49.3 | 127.8 | 86.29 | 20.25 |
|  | ios46 | Index of services | Index (2020=100) | 35.6 | 144.1 | 77.79 | 24.58 |
|  | mur49 | Manufacturing utilization rate index | Index (2020=100) | 82.87 | 123.01 | 107.66 | 7.02 |
| Prices | cpi58 | Consumer price index | Index (2020=100) | -0.4 | 6.3 | 2.50 | 1.37 |
|  | csi59 | Consumer sentiment index | composite index (standardized, dimensionless) | 68.7 | 122.7 | 100.48 | 8.66 |
|  | cpl60 | Cost-of-living index | Index (2020=100) | 59.75 | 116.12 | 87.77 | 14.40 |
|  | rsi61 | Retail sales index | Index (2020=100) | 36.3 | 122.3 | 75.95 | 23.56 |
|  | msi63 | Automobile sales index | Index (2020=100) | 20.8 | 134.9 | 58.77 | 28.87 |
| Housing | hcp67 | Housing construction performance | housing units | 9282 | 157503 | 43552.24 | 25333.59 |
|  | eei70 | Estimated index of equipment investment | Index (2020=100) | 46.6 | 134.1 | 79.90 | 20.51 |
|  | dcr71 | Domestic construction orders received | million KRW | 2555388 | 33593356 | 9552350.42 | 5068636.95 |
| Population | npp66 | Number of people moving in population | thousand persons | 450 | 962 | 671.14 | 110.98 |
| Leisure | ntg72 | Number of tourists in Gungneungwon | thousand persons | 0 | 2912 | 835.12 | 405.84 |
