# Supplementary material for: Explainable machine learning framework with Granger-based temporal predictive validation for monitoring fiscal risk in national health insurance expenditure
Source: PLoS One. 2026 Oct 29;21(10):e0361904. doi: 10.1371/journal.pone.0361904 (PMC13719042; doi:10.1371/journal.pone.0361904)
Supplement: Supplementary file 2 [file pone.0361904.s002.docx]

**S2 Appendix B**

This appendix reports the complete out-of-sample forecasting results and residual autocorrelation diagnostics for the baseline, indicator-based ML, and temporal validation models used in the national health insurance expenditure (HIE) forecasting framework. Specifically, Tables B1–B3 present detailed evaluation metrics for total, medical institution, and pharmacy HIEs, respectively. For each expenditure component, forecasting performance is assessed using both hold-out test evaluation and rolling-origin evaluation (ROE) with an expanding window. Multiple error metrics—including mean absolute error (MAE), root mean squared error (RMSE), mean absolute percentage error (MAPE), symmetric mean absolute percentage error (SMAPE), mean absolute scaled error (MASE), and root mean squared logarithmic error (RMSLE)—are reported to capture different aspects of predictive accuracy. To quantify statistical uncertainty, 95% bootstrap confidence intervals are provided for both the test set and ROE-based performance measures.

In addition to forecasting accuracy, this appendix reports residual autocorrelation diagnostics requested for the time-series framework. Table B4 presents Ljung–Box residual autocorrelation diagnostics for the selected LightGBM explanatory models using both hold-out test residuals and ROE residuals. Table B5 presents Ljung–Box residual autocorrelation diagnostics for the lagged regression models underlying the Granger-type temporal predictive validation of SHapley Additive exPlanations (SHAP)-selected indicators. These diagnostics complement the condensed performance comparisons and temporal predictive validation results reported in the main text by evaluating whether residual temporal dependence remained after forecasting and lagged regression modeling. Therefore, the results provide a more comprehensive basis for assessing predictive performance, temporal adequacy, and the cautious interpretation of temporal monitoring signals within the proposed framework.

In addition, Table B6 reports the lag-search-adjusted sensitivity analysis for the Granger-type temporal predictive validation, whereas Table B7 reports the final optimized hyperparameters for the selected LightGBM explanatory models to improve reproducibility.

**Table B1. Out-of-sample forecasting performance of baseline and indicator-based models for total HIE (Test and ROE with 95% bootstrap confidence intervals).**

| Algorithms | Metric | Test | | | | ROE | | |
| --- | --- | --- | --- | --- | --- | --- | --- | --- |
|  |  | Test Value | Mean | 95% CI Lower | 95% CI Upper | ROE Mean | 95% CI Lower | 95% CI Upper |
| Persistence | MAE | 0.063046 | 0.063047 | 0.037673 | 0.091913 | 0.057236 | 0.042355 | 0.074818 |
|  | RMSE | 0.095548 | 0.092966 | 0.051919 | 0.131972 | 0.057236 | 0.042692 | 0.074416 |
|  | MAPE | 0.274742 | 0.274742 | 0.163806 | 0.409742 | 0.250474 | 0.186613 | 0.322977 |
|  | SMAPE | 0.274867 | 0.274867 | 0.160038 | 0.414980 | 0.250584 | 0.188531 | 0.325357 |
|  | MASE | 1.202802 | 1.199006 | 0.720646 | 1.778408 | 1.091941 | 0.803770 | 1.440096 |
|  | RMSLE | 0.002633 | 0.002634 | 0.001558 | 0.003933 | 0.002401 | 0.001813 | 0.003112 |
| Same-Month-Last-Year | MAE | 0.102877 | 0.102878 | 0.078439 | 0.130826 | 0.086170 | 0.069123 | 0.104828 |
|  | RMSE | 0.122927 | 0.122030 | 0.094371 | 0.157467 | 0.086170 | 0.069227 | 0.104997 |
|  | MAPE | 0.448335 | 0.448335 | 0.344772 | 0.559815 | 0.376799 | 0.296267 | 0.453961 |
|  | SMAPE | 0.449740 | 0.449740 | 0.338806 | 0.574151 | 0.377794 | 0.302223 | 0.464253 |
|  | MASE | 1.962690 | 1.958922 | 1.482313 | 2.473999 | 1.643947 | 1.313499 | 1.995722 |
|  | RMSLE | 0.004309 | 0.004309 | 0.003258 | 0.005438 | 0.003619 | 0.002900 | 0.004383 |
| Ridge | MAE | 0.104303 | 0.104303 | 0.079085 | 0.131405 | 0.055047 | 0.042431 | 0.071221 |
|  | RMSE | 0.125116 | 0.124572 | 0.098619 | 0.150120 | 0.055047 | 0.042763 | 0.071860 |
|  | MAPE | 0.455561 | 0.455562 | 0.343919 | 0.566986 | 0.240880 | 0.184287 | 0.309755 |
|  | SMAPE | 0.454412 | 0.454413 | 0.336658 | 0.576406 | 0.240947 | 0.187936 | 0.309529 |
|  | MASE | 1.989885 | 1.991631 | 1.462775 | 2.516217 | 1.050174 | 0.798583 | 1.365907 |
|  | RMSLE | 0.004354 | 0.004354 | 0.003238 | 0.005469 | 0.002308 | 0.001792 | 0.002974 |
| LASSO | MAE | 0.094392 | 0.094393 | 0.071154 | 0.118699 | 0.049732 | 0.037766 | 0.064651 |
|  | RMSE | 0.109794 | 0.109810 | 0.086941 | 0.133332 | 0.049732 | 0.037476 | 0.064769 |
|  | MAPE | 0.412021 | 0.412021 | 0.324791 | 0.513752 | 0.217567 | 0.164746 | 0.280770 |
|  | SMAPE | 0.411296 | 0.411296 | 0.320637 | 0.507513 | 0.217611 | 0.166429 | 0.285739 |
|  | MASE | 1.800812 | 1.804580 | 1.383205 | 2.239745 | 0.948784 | 0.726048 | 1.232286 |
|  | RMSLE | 0.003941 | 0.003941 | 0.003035 | 0.004942 | 0.002085 | 0.001572 | 0.002712 |
| ElasticNet | MAE | 0.095664 | 0.095665 | 0.071840 | 0.118957 | 0.050825 | 0.038389 | 0.066318 |
|  | RMSE | 0.113586 | 0.113455 | 0.087706 | 0.139366 | 0.050825 | 0.038700 | 0.067018 |
|  | MAPE | 0.417686 | 0.417686 | 0.318810 | 0.527179 | 0.222335 | 0.169405 | 0.292270 |
|  | SMAPE | 0.416890 | 0.416890 | 0.310716 | 0.527777 | 0.222406 | 0.168420 | 0.290674 |
|  | MASE | 1.825087 | 1.822172 | 1.366038 | 2.285811 | 0.969639 | 0.733568 | 1.269439 |
|  | RMSLE | 0.003994 | 0.003995 | 0.003061 | 0.005052 | 0.002131 | 0.001624 | 0.002755 |
| RF | MAE | 0.244503 | 0.244504 | 0.209687 | 0.278640 | 0.048379 | 0.036619 | 0.064347 |
|  | RMSE | 0.257639 | 0.256494 | 0.224591 | 0.291482 | 0.048379 | 0.036727 | 0.063549 |
|  | MAPE | 1.065759 | 1.065759 | 0.931211 | 1.207991 | 0.211487 | 0.160013 | 0.279664 |
|  | SMAPE | 1.072092 | 1.072093 | 0.929999 | 1.211739 | 0.211851 | 0.159424 | 0.276710 |
|  | MASE | 4.664624 | 4.657854 | 4.058108 | 5.270350 | 0.922971 | 0.692496 | 1.206184 |
|  | RMSLE | 0.010817 | 0.010271 | 0.008955 | 0.011676 | 0.002030 | 0.001529 | 0.002664 |
| GradientBoost | MAE | 0.264839 | 0.264840 | 0.226698 | 0.300109 | 0.049549 | 0.035985 | 0.066266 |
|  | RMSE | 0.280352 | 0.279504 | 0.246584 | 0.312789 | 0.049549 | 0.035731 | 0.065816 |
|  | MAPE | 1.154388 | 1.154389 | 0.994248 | 1.307764 | 0.216610 | 0.154635 | 0.291868 |
|  | SMAPE | 1.161894 | 1.161894 | 0.984355 | 1.318257 | 0.216922 | 0.155095 | 0.286492 |
|  | MASE | 5.052594 | 5.068394 | 4.329513 | 5.756459 | 0.945283 | 0.683448 | 1.259319 |
|  | RMSLE | 0.011778 | 0.011131 | 0.009565 | 0.012604 | 0.002078 | 0.001498 | 0.002755 |
| XGBoost | MAE | 0.245177 | 0.245177 | 0.207333 | 0.283766 | 0.051693 | 0.037848 | 0.068620 |
|  | RMSE | 0.264774 | 0.263341 | 0.226369 | 0.308000 | 0.051693 | 0.037640 | 0.067866 |
|  | MAPE | 1.068456 | 1.068457 | 0.888111 | 1.240763 | 0.226016 | 0.165018 | 0.298311 |
|  | SMAPE | 1.075145 | 1.075145 | 0.899633 | 1.255836 | 0.226284 | 0.167190 | 0.301680 |
|  | MASE | 4.677473 | 4.684265 | 3.953146 | 5.483288 | 0.986196 | 0.724231 | 1.301070 |
|  | RMSLE | 0.011118 | 0.010300 | 0.008738 | 0.011924 | 0.002168 | 0.001604 | 0.002872 |
| LightGBM | MAE | 0.226645 | 0.226645 | 0.189772 | 0.262708 | 0.047775 | 0.035589 | 0.061838 |
|  | RMSE | 0.243408 | 0.242308 | 0.207919 | 0.281067 | 0.047775 | 0.035219 | 0.063560 |
|  | MAPE | 0.987736 | 0.987737 | 0.828063 | 1.140107 | 0.208885 | 0.157033 | 0.272705 |
|  | SMAPE | 0.993386 | 0.993386 | 0.839324 | 1.160992 | 0.209141 | 0.155150 | 0.275531 |
|  | MASE | 4.323923 | 4.336906 | 3.632328 | 5.031439 | 0.911439 | 0.678835 | 1.194176 |
|  | RMSLE | 0.010215 | 0.009517 | 0.007948 | 0.010894 | 0.002004 | 0.001515 | 0.002656 |
| AdaBoost | MAE | 0.221386 | 0.221387 | 0.189504 | 0.254097 | 0.051018 | 0.039364 | 0.066455 |
|  | RMSE | 0.235077 | 0.234092 | 0.201818 | 0.267901 | 0.051018 | 0.038669 | 0.065131 |
|  | MAPE | 0.964910 | 0.964911 | 0.827791 | 1.113343 | 0.223106 | 0.169215 | 0.283129 |
|  | SMAPE | 0.970179 | 0.970180 | 0.829894 | 1.117534 | 0.223438 | 0.173481 | 0.287621 |
|  | MASE | 4.223594 | 4.215406 | 3.650665 | 4.828698 | 0.973317 | 0.749871 | 1.252498 |
|  | RMSLE | 0.009864 | 0.009295 | 0.008037 | 0.010522 | 0.002141 | 0.001638 | 0.002766 |
| CatBoost | MAE | 0.309357 | 0.309357 | 0.255585 | 0.356623 | 0.054876 | 0.041124 | 0.072313 |
|  | RMSE | 0.333483 | 0.333436 | 0.295805 | 0.372547 | 0.054876 | 0.041553 | 0.071055 |
|  | MAPE | 1.348335 | 1.348336 | 1.134870 | 1.541489 | 0.239816 | 0.177921 | 0.315892 |
|  | SMAPE | 1.358972 | 1.358972 | 1.155951 | 1.563403 | 0.240151 | 0.177306 | 0.314489 |
|  | MASE | 5.901891 | 5.916408 | 4.903245 | 6.833649 | 1.046919 | 0.787558 | 1.380004 |
|  | RMSLE | 0.014030 | 0.013018 | 0.010800 | 0.015111 | 0.002301 | 0.001724 | 0.003014 |

**Table B2.** Out-of-sample forecasting performance of the baseline and indicator-based models for medical institutions HIE (Test and ROE with 95% bootstrap confidence intervals).

| Algorithms | Metric | Test | | | | ROE | | |
| --- | --- | --- | --- | --- | --- | --- | --- | --- |
|  |  | Test Value | Mean | 95% CI Lower | 95% CI Upper | ROE Mean | 95% CI Lower | 95% CI Upper |
| Persistence | MAE | 0.077111 | 0.077111 | 0.046273 | 0.110919 | 0.068330 | 0.051716 | 0.086687 |
|  | RMSE | 0.114295 | 0.110880 | 0.062536 | 0.162436 | 0.068330 | 0.051356 | 0.087397 |
|  | MAPE | 0.339302 | 0.339303 | 0.204817 | 0.492376 | 0.301954 | 0.228273 | 0.385589 |
|  | SMAPE | 0.339486 | 0.339486 | 0.209152 | 0.512962 | 0.302097 | 0.225157 | 0.384873 |
|  | MASE | 1.183466 | 1.180853 | 0.711445 | 1.766122 | 1.048696 | 0.794361 | 1.381585 |
|  | RMSLE | 0.003251 | 0.003252 | 0.001995 | 0.004768 | 0.002893 | 0.002174 | 0.003761 |
| Same-Month-Last-Year | MAE | 0.109034 | 0.109035 | 0.081093 | 0.142328 | 0.091442 | 0.072566 | 0.112378 |
|  | RMSE | 0.132413 | 0.131001 | 0.097906 | 0.173374 | 0.091442 | 0.072369 | 0.112017 |
|  | MAPE | 0.479706 | 0.479707 | 0.354911 | 0.613740 | 0.403753 | 0.320849 | 0.493660 |
|  | SMAPE | 0.481296 | 0.481296 | 0.357374 | 0.622609 | 0.404894 | 0.322414 | 0.498427 |
|  | MASE | 1.673415 | 1.669715 | 1.235986 | 2.126698 | 1.403409 | 1.129793 | 1.730348 |
|  | RMSLE | 0.004609 | 0.004610 | 0.003392 | 0.006018 | 0.003877 | 0.003100 | 0.004760 |
| Ridge | MAE | 0.111387 | 0.111388 | 0.080115 | 0.144104 | 0.065414 | 0.048209 | 0.084930 |
|  | RMSE | 0.138737 | 0.136857 | 0.104515 | 0.168375 | 0.065414 | 0.048319 | 0.085483 |
|  | MAPE | 0.491347 | 0.491348 | 0.353483 | 0.653713 | 0.288985 | 0.215481 | 0.375842 |
|  | SMAPE | 0.490144 | 0.490144 | 0.337551 | 0.635306 | 0.289090 | 0.214235 | 0.380794 |
|  | MASE | 1.709524 | 1.693737 | 1.170477 | 2.218165 | 1.003948 | 0.752759 | 1.320648 |
|  | RMSLE | 0.004694 | 0.004695 | 0.003403 | 0.006136 | 0.002769 | 0.002107 | 0.003598 |
| LASSO | MAE | 0.100842 | 0.100843 | 0.071244 | 0.131996 | 0.060803 | 0.045291 | 0.079713 |
|  | RMSE | 0.126114 | 0.124946 | 0.092866 | 0.160547 | 0.060803 | 0.045627 | 0.078831 |
|  | MAPE | 0.444632 | 0.444632 | 0.315813 | 0.583782 | 0.268601 | 0.199540 | 0.353190 |
|  | SMAPE | 0.443859 | 0.443860 | 0.323030 | 0.591979 | 0.268671 | 0.200602 | 0.357402 |
|  | MASE | 1.547683 | 1.537453 | 1.083451 | 2.030141 | 0.933176 | 0.701239 | 1.235147 |
|  | RMSLE | 0.004251 | 0.004251 | 0.003023 | 0.005503 | 0.002573 | 0.001932 | 0.003362 |
| ElasticNet | MAE | 0.100753 | 0.100754 | 0.069821 | 0.135070 | 0.061070 | 0.045458 | 0.081046 |
|  | RMSE | 0.128911 | 0.127535 | 0.090428 | 0.164145 | 0.061070 | 0.044881 | 0.080221 |
|  | MAPE | 0.444276 | 0.444276 | 0.300884 | 0.594573 | 0.269745 | 0.202923 | 0.349952 |
|  | SMAPE | 0.443500 | 0.443500 | 0.303787 | 0.577865 | 0.269851 | 0.200610 | 0.356318 |
|  | MASE | 1.546319 | 1.550096 | 1.089334 | 2.032773 | 0.937265 | 0.697629 | 1.228657 |
|  | RMSLE | 0.004248 | 0.004248 | 0.002975 | 0.005693 | 0.002584 | 0.001935 | 0.003413 |
| RF | MAE | 0.242277 | 0.242277 | 0.207279 | 0.278378 | 0.056636 | 0.043226 | 0.073677 |
|  | RMSE | 0.257974 | 0.256261 | 0.220783 | 0.299248 | 0.056636 | 0.043140 | 0.073622 |
|  | MAPE | 1.066466 | 1.066466 | 0.925317 | 1.216942 | 0.250029 | 0.190300 | 0.324616 |
|  | SMAPE | 1.072939 | 1.072939 | 0.925773 | 1.226256 | 0.250486 | 0.189910 | 0.327504 |
|  | MASE | 3.718350 | 3.702752 | 3.195919 | 4.239056 | 0.869225 | 0.656044 | 1.123602 |
|  | RMSLE | 0.010932 | 0.010275 | 0.008851 | 0.011801 | 0.002399 | 0.001823 | 0.003097 |
| GradientBoost | MAE | 0.176773 | 0.176774 | 0.140922 | 0.213271 | 0.066638 | 0.050036 | 0.087476 |
|  | RMSE | 0.198512 | 0.198066 | 0.158767 | 0.242447 | 0.066638 | 0.049190 | 0.088969 |
|  | MAPE | 0.777701 | 0.777701 | 0.618673 | 0.949135 | 0.294119 | 0.216969 | 0.389542 |
|  | SMAPE | 0.781523 | 0.781523 | 0.636746 | 0.938299 | 0.294467 | 0.219359 | 0.383835 |
|  | MASE | 2.713037 | 2.700108 | 2.167405 | 3.286632 | 1.022728 | 0.755694 | 1.352874 |
|  | RMSLE | 0.008397 | 0.007484 | 0.006040 | 0.008979 | 0.002820 | 0.002055 | 0.003734 |
| XGBoost | MAE | 0.225284 | 0.225285 | 0.190810 | 0.265550 | 0.079211 | 0.060300 | 0.101412 |
|  | RMSE | 0.243528 | 0.242041 | 0.202459 | 0.291102 | 0.079211 | 0.059841 | 0.102582 |
|  | MAPE | 0.991528 | 0.991528 | 0.842736 | 1.162461 | 0.349869 | 0.263290 | 0.445497 |
|  | SMAPE | 0.997291 | 0.997291 | 0.839209 | 1.155809 | 0.350575 | 0.263560 | 0.451668 |
|  | MASE | 3.457560 | 3.460764 | 2.908820 | 4.118821 | 1.215699 | 0.901545 | 1.571690 |
|  | RMSLE | 0.010316 | 0.009550 | 0.008006 | 0.011138 | 0.003357 | 0.002552 | 0.004319 |
| LightGBM | MAE | 0.220038 | 0.220038 | 0.183463 | 0.260853 | 0.059130 | 0.043452 | 0.080253 |
|  | RMSE | 0.239815 | 0.239340 | 0.199776 | 0.281818 | 0.059130 | 0.043461 | 0.077754 |
|  | MAPE | 0.968346 | 0.968346 | 0.813647 | 1.146141 | 0.260993 | 0.192401 | 0.349674 |
|  | SMAPE | 0.973935 | 0.973935 | 0.813530 | 1.154747 | 0.261472 | 0.195228 | 0.351572 |
|  | MASE | 3.377038 | 3.373070 | 2.768767 | 4.000898 | 0.907492 | 0.669396 | 1.209379 |
|  | RMSLE | 0.010158 | 0.009327 | 0.007684 | 0.010937 | 0.002504 | 0.001825 | 0.003358 |
| AdaBoost | MAE | 0.208954 | 0.208955 | 0.174127 | 0.250594 | 0.061404 | 0.047442 | 0.077054 |
|  | RMSE | 0.226705 | 0.225576 | 0.188129 | 0.267598 | 0.061404 | 0.047416 | 0.078378 |
|  | MAPE | 0.919592 | 0.919592 | 0.767517 | 1.067107 | 0.271204 | 0.212506 | 0.349728 |
|  | SMAPE | 0.924584 | 0.924584 | 0.778072 | 1.071819 | 0.271627 | 0.213019 | 0.345704 |
|  | MASE | 3.206939 | 3.212435 | 2.707884 | 3.771523 | 0.942399 | 0.732850 | 1.206366 |
|  | RMSLE | 0.009599 | 0.008854 | 0.007465 | 0.010532 | 0.002601 | 0.002019 | 0.003295 |
| CatBoost | MAE | 0.289805 | 0.289805 | 0.238258 | 0.340664 | 0.074369 | 0.052959 | 0.099027 |
|  | RMSE | 0.317061 | 0.317102 | 0.270018 | 0.370562 | 0.074369 | 0.053867 | 0.099369 |
|  | MAPE | 1.275518 | 1.275519 | 1.040709 | 1.489116 | 0.328301 | 0.233003 | 0.437188 |
|  | SMAPE | 1.285311 | 1.285311 | 1.058636 | 1.509790 | 0.329097 | 0.241304 | 0.437700 |
|  | MASE | 4.447790 | 4.451305 | 3.649001 | 5.214420 | 1.141376 | 0.832519 | 1.521812 |
|  | RMSLE | 0.013460 | 0.012308 | 0.010024 | 0.014325 | 0.003152 | 0.002274 | 0.004166 |

**Table B3.** Out-of-sample forecasting performance of the baseline and indicator-based models for pharmacy HIE (Test and ROE with 95% bootstrap confidence intervals).

| Algorithms | Metric | Test | | | | ROE | | |
| --- | --- | --- | --- | --- | --- | --- | --- | --- |
|  |  | Test Value | Mean | 95% CI Lower | 95% CI Upper | ROE Mean | 95% CI Lower | 95% CI Upper |
| Persistence | MAE | 0.038465 | 0.038465 | 0.027352 | 0.052024 | 0.040405 | 0.031657 | 0.050082 |
|  | RMSE | 0.049432 | 0.048263 | 0.033032 | 0.069112 | 0.040405 | 0.031743 | 0.049895 |
|  | MAPE | 0.180349 | 0.180349 | 0.127723 | 0.242983 | 0.190301 | 0.149218 | 0.233766 |
|  | SMAPE | 0.180442 | 0.180442 | 0.128087 | 0.241257 | 0.190373 | 0.148996 | 0.235938 |
|  | MASE | 0.685269 | 0.682239 | 0.487943 | 0.928661 | 0.719821 | 0.565833 | 0.886865 |
|  | RMSLE | 0.001723 | 0.001724 | 0.001229 | 0.002377 | 0.001818 | 0.001438 | 0.002220 |
| Same-Month-Last-Year | MAE | 0.100726 | 0.100727 | 0.085758 | 0.115173 | 0.083830 | 0.071496 | 0.096181 |
|  | RMSE | 0.107473 | 0.107482 | 0.094204 | 0.119785 | 0.083830 | 0.071827 | 0.095813 |
|  | MAPE | 0.472309 | 0.472310 | 0.395892 | 0.538456 | 0.394354 | 0.335592 | 0.451539 |
|  | SMAPE | 0.473583 | 0.473583 | 0.399542 | 0.539506 | 0.395173 | 0.336805 | 0.450500 |
|  | MASE | 1.794463 | 1.795511 | 1.505599 | 2.050775 | 1.493446 | 1.275427 | 1.706700 |
|  | RMSLE | 0.004523 | 0.004523 | 0.003870 | 0.005216 | 0.003774 | 0.003222 | 0.004338 |
| Ridge | MAE | 0.088313 | 0.088314 | 0.064813 | 0.113624 | 0.053375 | 0.043066 | 0.065677 |
|  | RMSE | 0.109065 | 0.108621 | 0.082682 | 0.133940 | 0.053375 | 0.042413 | 0.065811 |
|  | MAPE | 0.414429 | 0.414429 | 0.301847 | 0.544982 | 0.251654 | 0.202583 | 0.304612 |
|  | SMAPE | 0.413944 | 0.413944 | 0.301020 | 0.536167 | 0.251561 | 0.202556 | 0.306453 |
|  | MASE | 1.573322 | 1.580328 | 1.167155 | 2.039234 | 0.950880 | 0.763194 | 1.157828 |
|  | RMSLE | 0.003954 | 0.003954 | 0.002853 | 0.005193 | 0.002402 | 0.001924 | 0.002960 |
| LASSO | MAE | 0.089151 | 0.089151 | 0.069815 | 0.107807 | 0.046316 | 0.037553 | 0.056582 |
|  | RMSE | 0.099882 | 0.099450 | 0.083845 | 0.115159 | 0.046316 | 0.036956 | 0.056107 |
|  | MAPE | 0.417730 | 0.417731 | 0.330223 | 0.497048 | 0.218359 | 0.177298 | 0.262687 |
|  | SMAPE | 0.418453 | 0.418454 | 0.333675 | 0.501332 | 0.218255 | 0.178130 | 0.267026 |
|  | MASE | 1.588249 | 1.582930 | 1.276222 | 1.907799 | 0.825125 | 0.666832 | 0.995064 |
|  | RMSLE | 0.003996 | 0.003997 | 0.003202 | 0.004846 | 0.002084 | 0.001675 | 0.002521 |
| ElasticNet | MAE | 0.080593 | 0.080593 | 0.063105 | 0.098759 | 0.046870 | 0.037665 | 0.057096 |
|  | RMSE | 0.091527 | 0.091003 | 0.074224 | 0.107047 | 0.046870 | 0.036851 | 0.057090 |
|  | MAPE | 0.377790 | 0.377790 | 0.292599 | 0.459888 | 0.220962 | 0.178629 | 0.269909 |
|  | SMAPE | 0.378141 | 0.378142 | 0.295697 | 0.459418 | 0.220899 | 0.175484 | 0.265994 |
|  | MASE | 1.435780 | 1.429229 | 1.129286 | 1.749934 | 0.835006 | 0.673676 | 1.024709 |
|  | RMSLE | 0.003611 | 0.003612 | 0.002843 | 0.004399 | 0.002110 | 0.001679 | 0.002565 |
| RF | MAE | 0.266419 | 0.266420 | 0.238349 | 0.292251 | 0.043300 | 0.034149 | 0.052750 |
|  | RMSE | 0.275374 | 0.275154 | 0.249998 | 0.298924 | 0.043300 | 0.034133 | 0.053064 |
|  | MAPE | 1.248023 | 1.248023 | 1.110027 | 1.372217 | 0.203671 | 0.160837 | 0.246910 |
|  | SMAPE | 1.256392 | 1.256393 | 1.121346 | 1.376406 | 0.203918 | 0.161200 | 0.247435 |
|  | MASE | 4.746321 | 4.749604 | 4.231137 | 5.216931 | 0.771390 | 0.610077 | 0.943453 |
|  | RMSLE | 0.012398 | 0.011998 | 0.010712 | 0.013204 | 0.001947 | 0.001534 | 0.002368 |
| GradientBoost | MAE | 0.275914 | 0.275915 | 0.249469 | 0.302448 | 0.049565 | 0.040119 | 0.059371 |
|  | RMSE | 0.284450 | 0.284123 | 0.258365 | 0.309220 | 0.049565 | 0.039971 | 0.058994 |
|  | MAPE | 1.292602 | 1.292603 | 1.167743 | 1.419955 | 0.233407 | 0.186339 | 0.281434 |
|  | SMAPE | 1.301537 | 1.301537 | 1.168313 | 1.431743 | 0.233467 | 0.188845 | 0.281261 |
|  | MASE | 4.915472 | 4.908289 | 4.390590 | 5.420719 | 0.883013 | 0.707006 | 1.062398 |
|  | RMSLE | 0.012811 | 0.012429 | 0.011206 | 0.013646 | 0.002230 | 0.001779 | 0.002680 |
| XGBoost | MAE | 0.343903 | 0.343904 | 0.293278 | 0.391914 | 0.048966 | 0.039224 | 0.059441 |
|  | RMSE | 0.363620 | 0.362350 | 0.318408 | 0.399353 | 0.048966 | 0.039438 | 0.059158 |
|  | MAPE | 1.610733 | 1.610733 | 1.388610 | 1.834966 | 0.230393 | 0.183442 | 0.276254 |
|  | SMAPE | 1.625359 | 1.625360 | 1.400043 | 1.858519 | 0.230697 | 0.182599 | 0.277731 |
|  | MASE | 6.126715 | 6.127856 | 5.218642 | 6.988988 | 0.872333 | 0.691191 | 1.049372 |
|  | RMSLE | 0.016411 | 0.015521 | 0.013459 | 0.017547 | 0.002203 | 0.001786 | 0.002684 |
| LightGBM | MAE | 0.200402 | 0.200402 | 0.163692 | 0.236880 | 0.048972 | 0.036886 | 0.066248 |
|  | RMSE | 0.221700 | 0.220016 | 0.187779 | 0.252672 | 0.048972 | 0.036710 | 0.063647 |
|  | MAPE | 0.938255 | 0.938255 | 0.751607 | 1.101271 | 0.230257 | 0.172249 | 0.303707 |
|  | SMAPE | 0.943669 | 0.943670 | 0.774835 | 1.125631 | 0.230706 | 0.174468 | 0.306655 |
|  | MASE | 3.570201 | 3.581049 | 2.867095 | 4.199559 | 0.872441 | 0.654856 | 1.150055 |
|  | RMSLE | 0.009970 | 0.009012 | 0.007244 | 0.010787 | 0.002203 | 0.001635 | 0.002900 |
| AdaBoost | MAE | 0.235407 | 0.235408 | 0.208854 | 0.260879 | 0.041309 | 0.033832 | 0.049246 |
|  | RMSE | 0.244121 | 0.244085 | 0.219905 | 0.266014 | 0.041309 | 0.033372 | 0.049637 |
|  | MAPE | 1.102704 | 1.102705 | 0.975547 | 1.220147 | 0.194483 | 0.157877 | 0.235778 |
|  | SMAPE | 1.109276 | 1.109276 | 0.987837 | 1.224851 | 0.194618 | 0.158755 | 0.236938 |
|  | MASE | 4.193831 | 4.189688 | 3.705970 | 4.592975 | 0.735935 | 0.600431 | 0.882020 |
|  | RMSLE | 0.010983 | 0.010594 | 0.009383 | 0.011625 | 0.001859 | 0.001511 | 0.002230 |
| CatBoost | MAE | 0.271707 | 0.271708 | 0.229347 | 0.308180 | 0.048433 | 0.038816 | 0.059093 |
|  | RMSE | 0.288998 | 0.288790 | 0.254034 | 0.322759 | 0.048433 | 0.038913 | 0.058741 |
|  | MAPE | 1.272475 | 1.272475 | 1.091436 | 1.457001 | 0.227854 | 0.181457 | 0.279028 |
|  | SMAPE | 1.281694 | 1.281694 | 1.089023 | 1.469657 | 0.228152 | 0.182137 | 0.279386 |
|  | MASE | 4.840529 | 4.854039 | 4.151484 | 5.536401 | 0.862834 | 0.693040 | 1.045182 |
|  | RMSLE | 0.013017 | 0.012240 | 0.010549 | 0.013963 | 0.002179 | 0.001751 | 0.002662 |

Residual autocorrelation diagnostics were conducted using the Ljung–Box test for the selected LightGBM explanatory models. Residuals from the 2022–2023 hold-out test period and ROE residuals were examined at 6- and 12-month lags. When significant residual autocorrelation was detected, the corresponding forecasting diagnostics were interpreted cautiously because residual dependence indicates that some temporal structure was not fully captured.

**Table B4. Ljung–Box residual autocorrelation diagnostics for the LightGBM explanatory models.**

| Category | Residual source | Evaluation period | Number of residuals | Ljung–Box lag | Q-statistic | p-value | Autocorrelation detected |
| --- | --- | --- | --- | --- | --- | --- | --- |
| Total HIE | LightGBM test residuals | 2022–2023 hold-out test period | 24 | 6 | 8.6954 | 0.1914 | No |
|  |  |  | 24 | 12 | 16.6111 | 0.1648 | No |
|  | LightGBM ROE residuals | 2020–2023 expanding-window ROE | 48 | 6 | 3.4372 | 0.7523 | No |
|  |  |  | 48 | 12 | 7.9114 | 0.7920 | No |
| Medical institution HIE | LightGBM test residuals | 2022–2023 hold-out test period | 24 | 6 | 2.9068 | 0.8204 | No |
|  |  |  | 24 | 12 | 7.8985 | 0.7930 | No |
|  | LightGBM ROE residuals | 2020–2023 expanding-window ROE | 48 | 6 | 4.2576 | 0.6419 | No |
|  |  |  | 48 | 12 | 10.1164 | 0.6058 | No |
| Pharmacy HIE | LightGBM test residuals | 2022–2023 hold-out test period | 24 | 6 | 32.8266 | 1.13E-05 | Yes |
|  |  |  | 24 | 12 | 35.4970 | 0.0004 | Yes |
|  | LightGBM ROE residuals | 2020–2023 expanding-window ROE | 48 | 6 | 3.2889 | 0.7718 | No |
|  |  |  | 48 | 12 | 9.3162 | 0.6757 | No |

Note: The Ljung–Box test evaluates the null hypothesis that residuals are independently distributed up to the specified lag. A p-value below 0.05 indicates statistically significant residual autocorrelation. Test residuals refer to the residuals from the 2022–2023 hold-out test period, whereas ROE residuals refer to the expanding-window rolling-origin evaluation. The diagnostics were conducted at 6- and 12-month lags to examine short-to-medium-term and annual residual autocorrelation in monthly HIE forecasts.

The Ljung–Box diagnostics showed no statistically significant residual autocorrelation for total HIE and medical institution HIE at either the 6- or 12-month lag in both the hold-out test and ROE residuals. For pharmacy HIE, significant residual autocorrelation was detected in the hold-out test residuals at both the 6- and 12-month lags, whereas no statistically significant residual autocorrelation was detected in the ROE residuals. Therefore, the pharmacy HIE hold-out test results should be interpreted cautiously, whereas the ROE diagnostics do not suggest persistent residual autocorrelation across the broader expanding-window evaluation period.

To further assess the time-series adequacy of the Granger-type temporal predictive validation, residual autocorrelation diagnostics were conducted for the lagged regression models reported in Table 11 (in the main manuscript). For each HIE component and SHAP-selected indicator, the selected lag reported in Table 11 was used to refit the corresponding transformed-series lagged regression model, and the resulting residuals were evaluated using the Ljung–Box test at 6- and 12-month lags. These diagnostics were used to determine whether residual temporal dependence remained after stationary transformation and lagged regression adjustment.

**Table B5. Ljung–Box residual autocorrelation diagnostics for lagged regression models used in Granger-type temporal predictive validation.**

| Category | Indicator | Selected Lag  (months) | Residual source | Number of residuals | Ljung–Box lag | Q-statistic | p-value | Autocorrelation detected |
| --- | --- | --- | --- | --- | --- | --- | --- | --- |
| Total HIE | ios46 | 12 | Granger-type lagged regression residuals | 275 | 6 | 3.0014 | 0.8087 | No |
|  |  |  |  | 275 | 12 | 8.4868 | 0.7460 | No |
|  | cpl60 | 7 | Granger-type lagged regression residuals | 280 | 6 | 6.5008 | 0.3695 | No |
|  |  |  |  | 280 | 12 | 11.5070 | 0.4860 | No |
|  | nlc26 | 1 | Granger-type lagged regression residuals | 286 | 6 | 34.6572 | 5.02E-06 | Yes |
|  |  |  |  | 286 | 12 | 51.0343 | 9.19E-07 | Yes |
|  | tir28 | 1 | Granger-type lagged regression residuals | 286 | 6 | 39.9618 | 4.63E-07 | Yes |
|  |  |  |  | 286 | 12 | 60.9144 | 1.54E-08 | Yes |
|  | fer16 | 5 | Granger-type lagged regression residuals | 282 | 6 | 1.5569 | 0.9556 | No |
|  |  |  |  | 282 | 12 | 15.0200 | 0.2403 | No |
| Medical institution HIE | cpl60 | 1 | Granger-type lagged regression residuals | 286 | 6 | 33.7229 | 7.61E-06 | Yes |
|  |  |  |  | 286 | 12 | 51.0170 | 9.25E-07 | Yes |
|  | ios46 | 10 | Granger-type lagged regression residuals | 277 | 6 | 4.7811 | 0.5722 | No |
|  |  |  |  | 277 | 12 | 22.4206 | 0.0331 | Yes |
|  | nlc26 | 1 | Granger-type lagged regression residuals | 286 | 6 | 36.4989 | 2.20E-06 | Yes |
|  |  |  |  | 286 | 12 | 54.8909 | 1.89E-07 | Yes |
|  | msi63 | 2 | Granger-type lagged regression residuals | 285 | 6 | 29.9872 | 3.95E-05 | Yes |
|  |  |  |  | 285 | 12 | 48.5279 | 2.53E-06 | Yes |
|  | fer16 | 5 | Granger-type lagged regression residuals | 282 | 6 | 13.2076 | 0.0399 | Yes |
|  |  |  |  | 282 | 12 | 37.5822 | 0.0002 | Yes |
| Pharmacy HIE | cpl60 | 11 | Granger-type lagged regression residuals | 276 | 6 | 50.8066 | 3.24E-09 | Yes |
|  |  |  |  | 276 | 12 | 107.4589 | 1.90E-17 | Yes |
|  | npp66 | 11 | Granger-type lagged regression residuals | 276 | 6 | 36.2842 | 2.43E-06 | Yes |
|  |  |  |  | 276 | 12 | 72.7683 | 9.68E-11 | Yes |
|  | nlc26 | 12 | Granger-type lagged regression residuals | 275 | 6 | 13.0275 | 0.0426 | Yes |
|  |  |  |  | 275 | 12 | 44.1470 | 1.44E-05 | Yes |
|  | nti54 | 12 | Granger-type lagged regression residuals | 275 | 6 | 16.5682 | 0.0110 | Yes |
|  |  |  |  | 275 | 12 | 53.7566 | 3.02E-07 | Yes |
|  | alu35 | 9 | Granger-type lagged regression residuals | 278 | 6 | 85.4092 | 2.72E-16 | Yes |
|  |  |  |  | 278 | 12 | 159.0561 | 8.18E-28 | Yes |

Note: Residual diagnostics were conducted for the lagged regression models underlying the Granger-type temporal predictive validation reported in Table 11 (in the main manuscript). The selected lag refers to the lag between 1 and 12 months that produced the smallest SSR_F-test p-value for each SHAP-selected indicator in Table 11, whereas the Ljung–Box lag refers to the diagnostic horizon used to test for residual autocorrelation. The Ljung–Box test evaluates the null hypothesis that residuals are independently distributed up to the specified lag. A p-value below 0.05 indicates statistically significant residual autocorrelation. The diagnostics were conducted at 6- and 12-month lags to examine short-to-medium-term and annual residual autocorrelation in the transformed-series lagged regression residuals.

The residual diagnostics showed heterogeneous patterns across HIE components. For total HIE, residual autocorrelation was detected only for selected indicators. By contrast, residual autocorrelation was more frequently detected for medical institution and pharmacy HIEs, indicating that some temporal dependence remained unexplained in the corresponding lagged regression models. Therefore, temporal predictive validation results with significant residual autocorrelation were interpreted cautiously as indicative monitoring signals rather than as robust causal or structural validation.

**Table B6. Lag-search adjusted sensitivity analysis for Granger-type temporal predictive validation.**

| Category | Indicator | Selected Lag  (months) | p-value | Lag-search adjusted  p-value | FDR after lag-search adjustment | Bonferroni after lag-search adjustment | Interpretation |
| --- | --- | --- | --- | --- | --- | --- | --- |
| Total HIE | ios46 | 12 | 1.20E-06 | 1.44E-05 | 7.20E-05 | 7.20E-05 | Retained after lag-search and category-level correction,  more stable exploratory monitoring signal |
|  | cpl60 | 7 | 0.0002 | 0.0023 | 0.0058 | 0.0115 | Retained after lag-search and category-level correction,  more stable exploratory monitoring signal |
|  | nlc26 | 1 | 0.0075 | 0.0899 | 0.1499 | 0.4496 | Attenuated after lag-search adjustment,  weaker post-hoc screening signal |
|  | tir28 | 1 | 0.1555 | 1.0000 | 1.0000 | 1.0000 | Not statistically supported |
|  | fer16 | 5 | 0.1952 | 1.0000 | 1.0000 | 1.0000 | Not statistically supported |
| Medical institution HIE | cpl60 | 1 | 3.76E-05 | 0.0005 | 0.0018 | 0.0023 | Retained after lag-search and category-level correction,  more stable exploratory monitoring signal |
|  | ios46 | 10 | 5.88E-05 | 0.0007 | 0.0018 | 0.0035 | Retained after lag-search and category-level correction,  more stable exploratory monitoring signal |
|  | nlc26 | 1 | 0.0117 | 0.1402 | 0.2337 | 0.7012 | Attenuated after lag-search adjustment,  weaker post-hoc screening signal |
|  | msi63 | 2 | 0.0551 | 0.6616 | 0.8270 | 1.0000 | Not statistically supported |
|  | fer16 | 5 | 0.6882 | 1.0000 | 1.0000 | 1.0000 | Not statistically supported |
| Pharmacy HIE | cpl60 | 11 | 1.77E-08 | 2.13E-07 | 1.06E-06 | 1.06E-06 | Retained after lag-search and category-level correction,  more stable exploratory monitoring signal |
|  | npp66 | 11 | 3.61E-06 | 4.33E-05 | 0.0001 | 0.0002 | Retained after lag-search and category-level correction,  more stable exploratory monitoring signal |
|  | nlc26 | 12 | 3.95E-05 | 0.0005 | 0.0008 | 0.0024 | Retained after lag-search and category-level correction,  more stable exploratory monitoring signal |
|  | nti54 | 12 | 0.1196 | 1.0000 | 1.0000 | 1.0000 | Not statistically supported |
|  | alu35 | 9 | 0.5083 | 1.0000 | 1.0000 | 1.0000 | Not statistically supported |

Note: The p-value column reports the minimum SSR_F-test p-value selected across lags 1–12 for each SHAP-selected indicator. The lag-search-adjusted p-value was calculated as min(p × 12, 1), reflecting a conservative Bonferroni adjustment for the 12 candidate lags. The FDR and Bonferroni values after lag-search adjustment were then calculated within the SHAP-selected test family for each expenditure category. Results are interpreted as exploratory monitoring signals rather than confirmatory evidence of robust lagged predictive relationships or causal effects.

**Table B7. Final optimized hyperparameters for the selected LightGBM explanatory models.**

| Target series | Hyperparameter | Optimized value |
| --- | --- | --- |
| Total HIE | n_estimators | 200 |
|  | learning_rate | 0.2 |
|  | num_leaves | 31 |
|  | max_depth | -1 |
|  | min_child_samples | 5 |
|  | subsample | 0.5 |
|  | colsample_bytree | 0.7 |
|  | seed | 19 |
| Medical institution HIE | n_estimators | 300 |
|  | learning_rate | 0.5 |
|  | num_leaves | 31 |
|  | max_depth | -1 |
|  | min_child_samples | 5 |
|  | subsample | 0.5 |
|  | colsample_bytree | 1.0 |
|  | seed | 19 |
| Pharmacy HIE | n_estimators | 100 |
|  | learning_rate | 0.5 |
|  | num_leaves | 20 |
|  | max_depth | 3 |
|  | min_child_samples | 5 |
|  | subsample | 0.5 |
|  | colsample_bytree | 0.6 |
|  | seed | 19 |

Note: Hyperparameters were selected using GridSearchCV with TimeSeriesSplit within the training period. The optimized LightGBM models were refitted on the full training period before out-of-sample evaluation. A max_depth value of -1 indicates no explicit maximum tree-depth constraint.
