# Supplementary material for: Explainable machine learning framework with Granger-based temporal predictive validation for monitoring fiscal risk in national health insurance expenditure
Source: PLoS One. 2026 Oct 29;21(10):e0361904. doi: 10.1371/journal.pone.0361904 (PMC13719042; doi:10.1371/journal.pone.0361904)
Supplement: Supplementary file 3 [file pone.0361904.s003.docx]

**S3 Appendix C**

This appendix presents the complete SHapley Additive exPlanations (SHAP)-based feature importance results derived from the light gradient boosting machine (LightGBM) model for national health insurance expenditure (HIE). Specifically, Figs C1–C3 illustrate the top 20 features ranked by the mean absolute SHAP value for total, medical institution, and pharmacy HIEs, respectively.

For each expenditure component, the SHAP rankings summarize the relative contributions of national statistical indicators to model predictions, providing a transparent and comparable representation of indicator importance across provider types. These figures complement the selective discussion of key indicators in the main text and enable a comprehensive examination of the explanatory structure underlying the indicator-based ML models.

**Fig C1. Top 20 features ranked by mean |SHAP| value based on the LightGBM model for total HIE.**

**Fig C2. Top 20 features by ranked mean |SHAP| value based on the LightGBM model for medical institution HIE.**

**Fig C3. Top 20 features ranked by mean |SHAP| value based on the LightGBM model for pharmacy HIE.**

To examine whether the feature-importance rankings derived from the LightGBM–SHAP analysis were sensitive to model specification, we compared the LightGBM mean absolute SHAP rankings with the ElasticNet standardized absolute coefficient rankings. ElasticNet was used as a regularized linear comparator because it combines L1 and L2 penalties and is suitable for evaluating coefficient-based importance in the presence of correlated macroeconomic predictors. The comparison was summarized using top-5 overlap and Spearman’s rank correlation. This analysis was not intended to confirm the robustness of LightGBM–SHAP rankings but to assess the extent to which the identified indicators were dependent on the modeling specification. As listed in Table C4, agreement between the LightGBM–SHAP and ElasticNet coefficient rankings was limited for total and medical institution HIEs and partial for pharmacy HIE. These findings indicate that individual feature-importance rankings are model-dependent under correlated macroeconomic indicators. Therefore, the LightGBM-SHAP results should be interpreted as model-conditional, nonlinear explanatory signals rather than as definitive evidence for variable selection.

**Table C4. Category-level agreement between LightGBM–SHAP and ElasticNet coefficient rankings.**

| Category | Comparison | Top-5 overlap | Spearman’s ρ | Interpretation |
| --- | --- | --- | --- | --- |
| Total HIE | LightGBM–SHAP vs ElasticNet coefficient | 1/5 | 0.08123 | Limited agreement |
| Medical institution HIE |  | 1/5 | 0.18910 | Limited agreement |
| Pharmacy HIE |  | 2/5 | 0.22677 | Partial agreement |

**Table C5. Indicator-level comparison of LightGBM–SHAP top-five indicators with ElasticNet coefficient rankings.**

| Category | LightGBM–SHAP | | ElasticNet rank of the same indicator | Included in ElasticNet top-5 |
| --- | --- | --- | --- | --- |
|  | rank | indicator |  |  |
| Total HIE | 1 | Nlc26 | >5 | No |
|  | 2 | Cpl60 | >5 | No |
|  | 3 | Ios46 | 1 | Yes |
|  | 4 | Fer16 | >5 | No |
|  | 5 | Tir28 | >5 | No |
| Medical institution HIE | 1 | Cpl60 | >5 | No |
|  | 2 | Ios46 | 1 | Yes |
|  | 3 | Msi63 | >5 | No |
|  | 4 | Nlc26 | >5 | No |
|  | 5 | Fer16 | >5 | No |
| Pharmacy HIE | 1 | Nti54 | 2 | Yes |
|  | 2 | Cpl60 | >5 | No |
|  | 3 | Nlc26 | 1 | Yes |
|  | 4 | Npp66 | >5 | No |
|  | 5 | Alu35 | >5 | No |

To quantitatively assess predictor dependence and multicollinearity among the 60 national statistical indicators, pairwise Pearson correlations and variance inflation factor (VIF) diagnostics were conducted using the training-period predictor matrix after log1p preprocessing. These analyses were intended to characterize the extent of dependence among the macro-financial indicators and support the interpretation of SHAP-based feature importance under correlated predictors, rather than to perform additional feature elimination. Table C6 summarizes the pairwise correlation structure among the 60 indicators, including highly correlated indicator pairs and the correlation structure among the nine indicators subjected to Granger-type temporal predictive validation in Table 11 (in the main manuscript). Table C7 reports VIF diagnostics for all 60 indicators. Together, these analyses provide quantitative evidence supporting the interpretation of individual SHAP rankings as model-conditional and potentially cluster-level monitoring signals rather than as independent, variable-specific effects.

**Table C6. Pairwise correlation summary among the 60 national statistical indicators.**

**Panel A. Overall pairwise correlation summary.**

| Statistic | Value |
| --- | --- |
| No. of national statistical indicators | 60 |
| No. of observations (training period) | 228 |
| No. of pairwise correlations | 1,770 |
| Mean \|r\| | 0.5395 |
| Median \|r\| | 0.5684 |
| Maximum \|r\| | 0.9999 |
| No. of pairs with \|r\| ≥ 0.50 | 986 |
| No. of pairs with \|r\| ≥ 0.70 | 675 |
| No. of pairs with \|r\| ≥ 0.80 | 492 |
| No. of pairs with \|r\| ≥ 0.90 | 277 |
| Highest-correlation pair | aoi15 – iai52 (r = 0.9999) |

Note: Pairwise Pearson correlations were calculated using the training-period predictor matrix (n = 228) after log1p preprocessing. Autoregressive lag features were excluded so that the diagnostics reflected correlations among the 60 national statistical indicators only. More than half of all indicator pairs (986 of 1,770; 55.7%) exhibited moderate-to-strong correlations (|r| ≥ 0.50), and 277 pairs (15.6%) exceeded |r| = 0.90, indicating very high correlation. These results indicate that the 60 national statistical indicators are not mutually independent but instead form extensively overlapping conceptual clusters (e.g., growth-, price-, and global-related indicator groups), consistent with the VIF diagnostics reported in Table C7. This pairwise correlation summary was not used for feature elimination; instead, it supports the cluster-level interpretation of SHAP-based importance rankings discussed in the main text, which is examined further among the Granger-type temporal predictive validation indicators in Panel C below.

**Panel B. Top 20 highly correlated indicator pairs.**

| Rank | Indicator 1 | Indicator 2 | Pearson r |
| --- | --- | --- | --- |
| 1 | aoi15 | iai52 | 0.9999 |
| 2 | brr31 | crr30 | 0.9984 |
| 3 | mpi20 | msi21 | 0.9982 |
| 4 | eap3 | noe4 | 0.9975 |
| 5 | aoe14 | eai51 | 0.9972 |
| 6 | tir28 | tir29 | 0.9950 |
| 7 | rsi61 | wri45 | 0.9921 |
| 8 | kos40 | mcs27 | 0.9907 |
| 9 | ios46 | rsi61 | 0.9901 |
| 10 | iip19 | ios46 | 0.9892 |
| 11 | aoe14 | aoi15 | 0.9874 |
| 12 | aoe14 | iai52 | 0.9866 |
| 13 | aoi15 | eai51 | 0.9863 |
| 14 | eai51 | iai52 | 0.9858 |
| 15 | ios46 | wri45 | 0.9855 |
| 16 | lir47 | rer48 | 0.9854 |
| 17 | cpl60 | evi56 | 0.9853 |
| 18 | cpl60 | ios46 | 0.9831 |
| 19 | evi56 | ios46 | 0.9828 |
| 20 | evi56 | ivi55 | 0.9810 |

Note: All pairs listed exceed |r| = 0.90 (very high correlation).

**Panel C. Pairwise correlations among the nine indicators subjected to Granger-type temporal predictive validation (Table 11 in the main manuscript).**

| Rank | Indicator 1 | Indicator 2 | Pearson r | Correlation category |
| --- | --- | --- | --- | --- |
| 1 | cpl60 | ios46 | 0.9831 | Very high (\|r\| ≥ 0.90) |
| 2 | cpl60 | fer16 | 0.9735 | Very high |
| 3 | fer16 | ios46 | 0.9574 | Very high |
| 4 | ios46 | nlc26 | 0.9496 | Very high |
| 5 | cpl60 | nti54 | −0.9420 | Very high |
| 6 | cpl60 | nlc26 | 0.9323 | Very high |
| 7 | fer16 | nlc26 | 0.9273 | Very high |
| 8 | ios46 | msi63 | 0.9164 | Very high |
| 9 | fer16 | nti54 | −0.9071 | Very high |
| 10 | ios46 | nti54 | −0.8976 | High (0.70 ≤ \|r\| < 0.90) |
| 11 | msi63 | tir28 | −0.8696 | High |
| 12 | cpl60 | msi63 | 0.8640 | High |
| 13 | ios46 | tir28 | −0.8516 | High |
| 14 | msi63 | nlc26 | 0.8388 | High |
| 15 | nlc26 | nti54 | −0.8332 | High |
| 16 | fer16 | msi63 | 0.8286 | High |
| 17 | cpl60 | tir28 | −0.8175 | High |
| 18 | fer16 | tir28 | −0.7982 | High |
| 19 | nlc26 | tir28 | −0.7951 | High |
| 20 | msi63 | nti54 | −0.7237 | High |
| 21 | cpl60 | npp66 | −0.6283 | Moderate (0.50 ≤ \|r\| < 0.70) |
| 22 | npp66 | tir28 | 0.6283 | Moderate |
| 23 | ios46 | npp66 | −0.6239 | Moderate |
| 24 | nti54 | tir28 | 0.6149 | Moderate |
| 25 | fer16 | npp66 | −0.5915 | Moderate |
| 26 | msi63 | npp66 | −0.5654 | Moderate |
| 27 | nlc26 | npp66 | −0.5614 | Moderate |
| 28 | npp66 | nti54 | 0.5559 | Moderate |
| 29 | alu35 | nti54 | −0.4440 | Lower (\|r\| < 0.50) |
| 30 | alu35 | fer16 | 0.4087 | Lower |
| 31 | alu35 | nlc26 | 0.3184 | Lower |
| 32 | alu35 | cpl60 | 0.3143 | Lower |
| 33 | alu35 | ios46 | 0.2522 | Lower |
| 34 | alu35 | tir28 | 0.1037 | Lower |
| 35 | alu35 | msi63 | 0.0673 | Lower |
| 36 | alu35 | npp66 | −0.0239 | Lower |

Note: These nine indicators comprise the union of all indicators subjected to Granger-type temporal predictive validation in Table 11 (total, medical institution, and pharmacy HIE combined). Among the 36 pairwise correlations, cpl60, ios46, fer16, and nlc26 form a tightly correlated cluster (|r| = 0.93–0.98), and msi63 and tir28 are highly correlated with this cluster (|r| = 0.80–0.92). By contrast, alu35 shows uniformly low correlation with all other indicators (|r| ≤ 0.44), whereas npp66 shows moderate correlations with seven of the remaining eight indicators (|r| = 0.56–0.63) and almost no correlation with alu35 (|r| = 0.02). These patterns indicate comparatively distinct correlation structures among the pharmacy-related SHAP-selected indicators; however, of alu35 and npp66, only npp66 showed exploratory lagged monitoring support in Table 11, whereas alu35 did not.

**Table C7. VIF diagnostics for national statistical indicators.**

**Panel A. VIF summary.**

| Statistic | Value |
| --- | --- |
| No. of indicators evaluated | 60 |
| No. of observations (training period) | 228 |
| No. of indicators with VIF > 5 | 60 |
| No. of indicators with VIF > 10 | 52 |
| No. of indicators with infinite VIF | 0 |
| Median VIF | 197.23 |
| Maximum finite VIF | 10,897,589.74 |
| Highest-VIF indicator | eai51 (VIF = 10,897,589.74) |

Note: VIF diagnostics were calculated using the training-period predictor matrix (n = 228) after log1p preprocessing. Autoregressive lag features were excluded so that the diagnostics reflected multicollinearity among the 60 national statistical indicators only. For VIF calculation, the indicator matrix was standardized to improve numerical stability; because VIF is scale-invariant, standardization does not alter the resulting VIF values. All 60 indicators exceeded the conventional VIF threshold of 5, and 52 of 60 indicators (86.7%) had VIF > 10, with a median VIF of 197.23 and a maximum finite VIF of 10,897,589.74 (eai51). This indicates that several indicators exhibit near-perfect linear dependency on the remaining predictors. These findings are consistent with the pairwise correlation summary reported in Table C6 and indicate that the 60 indicators cannot be treated as approximately independent predictors. This VIF summary was not used for feature elimination; instead, it supports the cluster-level interpretation of SHAP-based importance rankings examined further at the indicator level in Panel B below.

**Panel B. Indicator-level VIF diagnostics.**

| Rank | Indicator | R² (auxiliary regression) | VIF | VIF category |
| --- | --- | --- | --- | --- |
| 1 | eai51 | 0.9999999 | 10,897,589.74 | High (VIF > 10) |
| 2 | iai52 | 0.9999999 | 10,196,555.83 | High |
| 3 | itt53 | 0.9999997 | 3,491,263.75 | High |
| 4 | ivi55 | 0.9999997 | 3,431,507.51 | High |
| 5 | evi56 | 0.9999997 | 2,895,031.40 | High |
| 6 | **nti54** | 0.9999983 | 577,249.16 | High |
| 7 | noe4 | 0.9999738 | 38,115.72 | High |
| 8 | eap3 | 0.9999729 | 36,878.73 | High |
| 9 | aoi15 | 0.9999120 | 11,361.35 | High |
| 10 | mcs27 | 0.9997541 | 4,065.88 | High |
| 11 | **ios46** | 0.9994954 | 1,981.85 | High |
| 12 | kos40 | 0.9994868 | 1,948.37 | High |
| 13 | **cpl60** | 0.9992858 | 1,400.24 | High |
| 14 | msi21 | 0.9991963 | 1,244.24 | High |
| 15 | mpi20 | 0.9991830 | 1,224.04 | High |
| 16 | yea7 | 0.9989265 | 931.57 | High |
| 17 | wri45 | 0.9989208 | 926.60 | High |
| 18 | iip19 | 0.9988879 | 899.23 | High |
| 19 | **tir28** | 0.9988570 | 874.87 | High |
| 20 | rsi61 | 0.9987959 | 830.47 | High |
| 21 | **fer16** | 0.9986089 | 718.85 | High |
| 22 | crr30 | 0.9985002 | 666.77 | High |
| 23 | tir29 | 0.9982597 | 574.63 | High |
| 24 | lir47 | 0.9980870 | 522.75 | High |
| 25 | brr31 | 0.9980641 | 516.55 | High |
| 26 | aoe14 | 0.9979338 | 483.98 | High |
| 27 | rer48 | 0.9976916 | 433.21 | High |
| 28 | yer9 | 0.9970167 | 335.20 | High |
| 29 | wer5 | 0.9966591 | 299.32 | High |
| 30 | mii22 | 0.9950551 | 202.23 | High |
| 31 | wur6 | 0.9947982 | 192.24 | High |
| 32 | gol44 | 0.9947891 | 191.90 | High |
| 33 | eer1 | 0.9930159 | 143.18 | High |
| 34 | **nlc26** | 0.9918108 | 122.11 | High |
| 35 | tin38 | 0.9874767 | 79.85 | High |
| 36 | iop43 | 0.9846344 | 65.08 | High |
| 37 | lea36 | 0.9845684 | 64.80 | High |
| 38 | nbd34 | 0.9845201 | 64.60 | High |
| 39 | **msi63** | 0.9787782 | 47.12 | High |
| 40 | yur8 | 0.9763285 | 42.24 | High |
| 41 | bdr32 | 0.9756433 | 41.06 | High |
| 42 | eei70 | 0.9755690 | 40.93 | High |
| 43 | zin37 | 0.9660435 | 29.45 | High |
| 44 | mur49 | 0.9636698 | 27.53 | High |
| 45 | kod41 | 0.9616406 | 26.07 | High |
| 46 | nic39 | 0.9480675 | 19.26 | High |
| 47 | wer17 | 0.9435204 | 17.71 | High |
| 48 | abd33 | 0.9404039 | 16.78 | High |
| 49 | ccl25 | 0.9393761 | 16.50 | High |
| 50 | mcu42 | 0.9379281 | 16.11 | High |
| 51 | cpi58 | 0.9118570 | 11.35 | High |
| 52 | ccc24 | 0.9053126 | 10.56 | High |
| 53 | eur2 | 0.8939387 | 9.43 | Elevated (5 < VIF ≤ 10) |
| 54 | **alu35** | 0.8831086 | 8.56 | Elevated |
| 55 | dcr71 | 0.8581149 | 7.05 | Elevated |
| 56 | csi59 | 0.8563055 | 6.96 | Elevated |
| 57 | ntg72 | 0.8281483 | 5.82 | Elevated |
| 58 | opo23 | 0.8248061 | 5.71 | Elevated |
| 59 | **npp66** | 0.8227517 | 5.64 | Elevated |
| 60 | hcp67 | 0.8061006 | 5.16 | Elevated |

Note: Bold entries (nti54, ios46, cpl60, tir28, fer16, nlc26, msi63, alu35, npp66) represent the nine indicators subjected to Granger-type temporal predictive validation (Table 11 in the main manuscript). Among these nine indicators, VIF values ranged widely, from 5.64 (npp66) to 577,249.16 (nti54), indicating that the tested indicators—including those showing exploratory lagged monitoring support in Table 11—differ substantially in the extent to which they are linearly explained by the remaining 59 indicators. This indicator-level pattern, together with the overall summary in Panel A, supports interpreting the SHAP-based importance rankings and their downstream Granger-type temporal screening results as model-conditional, cluster-level monitoring signals rather than as evidence of independent, variable-specific effects.
