# Supplementary material for: Explainable machine learning framework with Granger-based temporal predictive validation for monitoring fiscal risk in national health insurance expenditure
Source: PLoS One. 2026 Oct 29;21(10):e0361904. doi: 10.1371/journal.pone.0361904 (PMC13719042; doi:10.1371/journal.pone.0361904)
Supplement: Supplementary file 4 [file pone.0361904.s004.docx]

**S4 Appendix D**

This appendix reports the stationarity diagnostics for the 60 national statistical indicators used as common explanatory variables across the models for total, medical institution, and pharmacy HIEs . The stationarity of each indicator was assessed using both the augmented Dickey–Fuller (ADF) test and the Kwiatkowski–Phillips–Schmidt–Shin (KPSS) test. The ADF test evaluates the null hypothesis of a unit root, whereas the KPSS test evaluates the null hypothesis of stationarity. Because these tests assess the time-series properties of each individual indicator, the results are reported for the common explanatory-variable set rather than separately by HIE outcome.

For each indicator, Table D1 reports the ADF and KPSS results in levels, the transformation applied where necessary, and the corresponding stationarity results after transformation. Indicators with nonnegative values were transformed using first-order log1p differencing, whereas indicators containing negative values were transformed using first differencing. These diagnostics were used to determine the stationarity-transformed series used in the Granger-type temporal predictive validation analysis. Indicators that remained mixed or inconclusive after transformation were not considered fully stationary; instead, results involving these indicators were retained as supplementary screening evidence and interpreted cautiously in the main text.

**Table D1. Stationarity diagnostics for the 60 national statistical indicators used as common explanatory variables across all HIE models.**

| Feature | Level ADF | | Level KPSS | | Level | Transformation | Transformed ADF | | Transformed KPSS | | Transformed Stationarity |
| --- | --- | --- | --- | --- | --- | --- | --- | --- | --- | --- | --- |
|  | Statistic | p-value | Statistic | p-value | Stationarity |  | Statistic | p-value | Statistic | p-value |  |
| eer1 | −0.6319 | 0.8636 | 2.6424 | 0.0100 | Nonstationary | log-diff | −5.4424 | 2.76E-06 | 0.0846 | 0.1000 | Stationary |
| eur2 | −3.1957 | 0.0202 | 0.6169 | 0.0211 | Mixed or inconclusive | log-diff | −3.7803 | 0.0031 | 0.1335 | 0.1000 | Stationary |
| eap3 | −0.5308 | 0.8859 | 2.7241 | 0.0100 | Nonstationary | log-diff | −4.2137 | 0.0006 | 0.0969 | 0.1000 | Stationary |
| noe4 | −0.5197 | 0.8881 | 2.7143 | 0.0100 | Nonstationary | log-diff | −4.1984 | 0.0007 | 0.1124 | 0.1000 | Stationary |
| wer5 | −0.7443 | 0.8349 | 2.3668 | 0.0100 | Nonstationary | log-diff | −3.7661 | 0.0033 | 0.1012 | 0.1000 | Stationary |
| wur6 | −4.0272 | 0.0013 | 0.3654 | 0.0921 | Stationary | log-diff | −3.6423 | 0.0050 | 0.1963 | 0.1000 | Stationary |
| yea7 | −2.0251 | 0.2756 | 2.0840 | 0.0100 | Nonstationary | log-diff | −4.3572 | 0.0004 | 0.2361 | 0.1000 | Stationary |
| yur8 | −1.7146 | 0.4236 | 0.3548 | 0.0966 | Mixed or inconclusive | log-diff | −4.8275 | 4.82E-05 | 0.0347 | 0.1000 | Stationary |
| yer9 | −1.3695 | 0.5968 | 0.5709 | 0.0257 | Nonstationary | log-diff | −3.8666 | 0.0023 | 0.1770 | 0.1000 | Stationary |
| aoe14 | −1.3891 | 0.5875 | 2.4021 | 0.0100 | Nonstationary | log-diff | −4.0914 | 0.0010 | 0.1116 | 0.1000 | Stationary |
| aoi15 | −1.4045 | 0.5801 | 2.2290 | 0.0100 | Nonstationary | log-diff | −4.6820 | 9.09E-05 | 0.1016 | 0.1000 | Stationary |
| fer16 | −1.8215 | 0.3698 | 2.6239 | 0.0100 | Nonstationary | log-diff | −6.0300 | 1.42E-07 | 0.8350 | 0.0100 | Mixed or inconclusive |
| wer17 | −2.3502 | 0.1563 | 0.2989 | 0.1000 | Mixed or inconclusive | log-diff | −13.6810 | 1.40E-25 | 0.0498 | 0.1000 | Stationary |
| eai51 | −1.2866 | 0.6353 | 2.4433 | 0.0100 | Nonstationary | log-diff | −4.7134 | 7.94E-05 | 0.0902 | 0.1000 | Stationary |
| iai52 | −1.2846 | 0.6362 | 2.2187 | 0.0100 | Nonstationary | log-diff | −4.6340 | 0.0001 | 0.0963 | 0.1000 | Stationary |
| itt53 | −1.1946 | 0.6759 | 2.6122 | 0.0100 | Nonstationary | log-diff | −4.9771 | 2.46E-05 | 0.0661 | 0.1000 | Stationary |
| nti54 | −2.9001 | 0.0453 | 2.0834 | 0.0100 | Mixed or inconclusive | log-diff | −6.3875 | 2.14E-08 | 0.3648 | 0.0923 | Stationary |
| ivi55 | −0.9427 | 0.7736 | 2.6398 | 0.0100 | Nonstationary | log-diff | −5.0795 | 1.54E-05 | 0.0216 | 0.1000 | Stationary |
| evi56 | −0.8820 | 0.7939 | 2.6890 | 0.0100 | Nonstationary | log-diff | −4.4852 | 0.0002 | 0.2819 | 0.1000 | Stationary |
| tir28 | −2.1683 | 0.2179 | 2.0568 | 0.0100 | Nonstationary | log-diff | −12.1436 | 1.63E-22 | 0.2940 | 0.1000 | Stationary |
| tir29 | −2.0541 | 0.2634 | 2.1027 | 0.0100 | Nonstationary | log-diff | −12.7356 | 9.17E-24 | 0.3031 | 0.1000 | Stationary |
| crr30 | −2.0907 | 0.2483 | 1.8450 | 0.0100 | Nonstationary | log-diff | −6.4486 | 1.54E-08 | 0.1687 | 0.1000 | Stationary |
| brr31 | −2.1164 | 0.2380 | 1.8331 | 0.0100 | Nonstationary | log-diff | −5.5290 | 1.81E-06 | 0.1605 | 0.1000 | Stationary |
| lir47 | −2.3357 | 0.1607 | 2.1078 | 0.0100 | Nonstationary | log-diff | −3.2233 | 0.0187 | 0.2944 | 0.1000 | Stationary |
| rer48 | −2.6416 | 0.0847 | 1.8795 | 0.0100 | Nonstationary | log-diff | −8.4140 | 2.07E-13 | 0.2605 | 0.1000 | Stationary |
| iip19 | −1.4341 | 0.5658 | 2.6595 | 0.0100 | Nonstationary | log-diff | −4.0220 | 0.0013 | 0.0227 | 0.1000 | Stationary |
| mpi20 | −1.8668 | 0.3478 | 2.3961 | 0.0100 | Nonstationary | log-diff | −4.6753 | 9.36E-05 | 0.2998 | 0.1000 | Stationary |
| msi21 | −1.9600 | 0.3044 | 2.3866 | 0.0100 | Nonstationary | log-diff | −4.9645 | 2.61E-05 | 0.5000 | 0.0417 | Mixed or inconclusive |
| mii22 | −1.6973 | 0.4325 | 2.6374 | 0.0100 | Nonstationary | log-diff | −4.0634 | 0.0011 | 0.4272 | 0.0654 | Stationary |
| opo23 | −1.6703 | 0.4465 | 1.3748 | 0.0100 | Nonstationary | log-diff | −7.1241 | 3.66E-10 | 0.0326 | 0.1000 | Stationary |
| alu35 | −3.3362 | 0.0133 | 0.4191 | 0.0689 | Stationary | log-diff | −14.8815 | 1.61E-27 | 0.0310 | 0.1000 | Stationary |
| lea36 | −2.3389 | 0.1597 | 1.5703 | 0.0100 | Nonstationary | log-diff | −13.3476 | 5.75E-25 | 0.1651 | 0.1000 | Stationary |
| zin37 | −2.2223 | 0.1983 | 1.2671 | 0.0100 | Nonstationary | log-diff | −12.8232 | 6.09E-24 | 0.0559 | 0.1000 | Stationary |
| tin38 | −2.3699 | 0.1504 | 1.8686 | 0.0100 | Nonstationary | log-diff | −8.7371 | 3.08E-14 | 0.0627 | 0.1000 | Stationary |
| nic39 | −2.5151 | 0.1119 | 0.2715 | 0.1000 | Mixed or inconclusive | log-diff | −12.6704 | 1.25E-23 | 0.0702 | 0.1000 | Stationary |
| iop43 | −2.7158 | 0.0713 | 0.8210 | 0.0100 | Nonstationary | log-diff | −9.7062 | 1.04E-16 | 0.0758 | 0.1000 | Stationary |
| gol44 | −0.4640 | 0.8989 | 2.3083 | 0.0100 | Nonstationary | log-diff | −14.6835 | 3.13E-27 | 0.2476 | 0.1000 | Stationary |
| ccc24 | −4.3469 | 0.0004 | 0.2201 | 0.1000 | Stationary | log-diff | −5.2177 | 8.09E-06 | 0.0181 | 0.1000 | Stationary |
| ccl25 | −4.0509 | 0.0012 | 0.0472 | 0.1000 | Stationary | log-diff | −5.8593 | 3.44E-07 | 0.0651 | 0.1000 | Stationary |
| nlc26 | 0.8717 | 0.9927 | 2.4794 | 0.0100 | Nonstationary | log-diff | −3.3181 | 0.0141 | 0.4776 | 0.0467 | Mixed or inconclusive |
| mcs27 | −0.2879 | 0.9272 | 2.5394 | 0.0100 | Nonstationary | log-diff | −16.5347 | 2.00E-29 | 0.0459 | 0.1000 | Stationary |
| bdr32 | −5.2171 | 8.11E-06 | 1.2882 | 0.0100 | Mixed or inconclusive | log-diff | −5.5949 | 1.30E-06 | 0.3965 | 0.0787 | Stationary |
| abd33 | −6.7936 | 2.33E-09 | 1.2174 | 0.0100 | Mixed or inconclusive | log-diff | −4.9188 | 3.21E-05 | 0.2589 | 0.1000 | Stationary |
| nbd34 | −2.2676 | 0.1826 | 2.3940 | 0.0100 | Nonstationary | log-diff | −5.4053 | 3.30E-06 | 0.0046 | 0.1000 | Stationary |
| kos40 | −1.0839 | 0.7215 | 2.3896 | 0.0100 | Nonstationary | log-diff | −16.9107 | 1.01E-29 | 0.0501 | 0.1000 | Stationary |
| kod41 | −5.1982 | 8.87E-06 | 0.4148 | 0.0708 | Stationary | log-diff | −17.2577 | 5.97E-30 | 0.2960 | 0.1000 | Stationary |
| mcu42 | −3.7408 | 0.0036 | 1.3833 | 0.0100 | Mixed or inconclusive | log-diff | −7.7584 | 9.60E-12 | 0.0197 | 0.1000 | Stationary |
| wri45 | −0.5336 | 0.8853 | 2.6215 | 0.0100 | Nonstationary | log-diff | −3.7421 | 0.0036 | 0.1171 | 0.1000 | Stationary |
| ios46 | 0.8292 | 0.9921 | 2.6610 | 0.0100 | Nonstationary | log-diff | −3.6852 | 0.0043 | 0.0822 | 0.1000 | Stationary |
| mur49 | −1.8308 | 0.3653 | 1.5970 | 0.0100 | Nonstationary | log-diff | −5.8995 | 2.80E-07 | 0.1752 | 0.1000 | Stationary |
| cpi58 | −2.2908 | 0.1749 | 0.5220 | 0.0367 | Nonstationary | First differencing | −5.5427 | 1.69E-06 | 0.0445 | 0.1000 | Stationary |
| csi59 | −5.3263 | 4.83E-06 | 0.1181 | 0.1000 | Stationary | log-diff | −7.7981 | 7.62E-12 | 0.0182 | 0.1000 | Stationary |
| cpl60 | −0.1177 | 0.9476 | 2.6136 | 0.0100 | Nonstationary | log-diff | −3.2646 | 0.0165 | 0.3998 | 0.0772 | Stationary |
| rsi61 | 0.0783 | 0.9645 | 2.6989 | 0.0100 | Nonstationary | log-diff | −3.9111 | 0.0020 | 0.1868 | 0.1000 | Stationary |
| msi63 | 0.3397 | 0.9791 | 2.5885 | 0.0100 | Nonstationary | log-diff | −6.7528 | 2.92E-09 | 0.0435 | 0.1000 | Stationary |
| hcp67 | −3.0082 | 0.0341 | 0.2761 | 0.1000 | Stationary | log-diff | −5.7409 | 6.28E-07 | 0.0524 | 0.1000 | Stationary |
| eei70 | −0.7638 | 0.8295 | 2.5793 | 0.0100 | Nonstationary | log-diff | −5.0491 | 1.77E-05 | 0.0353 | 0.1000 | Stationary |
| dcr71 | −1.1129 | 0.7099 | 2.3704 | 0.0100 | Nonstationary | log-diff | −5.7558 | 5.82E-07 | 0.0943 | 0.1000 | Stationary |
| npp66 | −0.5926 | 0.8726 | 2.5682 | 0.0100 | Nonstationary | log-diff | −5.1117 | 1.33E-05 | 0.0639 | 0.1000 | Stationary |
| ntg72 | −2.5819 | 0.0968 | 1.4398 | 0.0100 | Nonstationary | log-diff | −10.6098 | 5.87E-19 | 0.2201 | 0.1000 | Stationary |

Note: ADF denotes the augmented Dickey–Fuller test, for which the null hypothesis is the presence of a unit root. KPSS denotes the Kwiatkowski–Phillips–Schmidt–Shin test, for which the null hypothesis is stationarity. Stationarity classifications were determined jointly using ADF and KPSS results at the 5% significance level. Transformed series were used for Granger-type temporal predictive validation where stationarity in levels was not supported. “Mixed or inconclusive” indicates that the ADF and KPSS diagnostics did not provide fully consistent evidence of stationarity. Therefore, Granger-type temporal predictive validation results involving indicators that remained mixed or inconclusive after transformation should be interpreted cautiously given incomplete stationarity support in the transformed series, rather than as fully robust temporal predictive evidence.
